# Cancer-associated fibroblasts reuse cancer-derived lactate to maintain a fibrotic and immunosuppressive microenvironment in pancreatic cancer

**DOI:** 10.1172/jci.insight.163022

**Published:** 2023-10-23

**Authors:** Fumimasa Kitamura, Takashi Semba, Noriko Yasuda-Yoshihara, Kosuke Yamada, Akiho Nishimura, Juntaro Yamasaki, Osamu Nagano, Tadahito Yasuda, Atsuko Yonemura, Yilin Tong, Huaitao Wang, Takahiko Akiyama, Kazuki Matsumura, Norio Uemura, Rumi Itoyama, Luke Bu, Lingfeng Fu, Xichen Hu, Feng Wei, Kosuke Mima, Katsunori Imai, Hiromitsu Hayashi, Yo-ichi Yamashita, Yuji Miyamoto, Hideo Baba, Takatsugu Ishimoto

**Affiliations:** 1 Department of Gastroenterological Surgery, Graduate School of Medical Sciences, and; 2 Gastrointestinal Cancer Biology, International Research Center for Medical Sciences, Kumamoto University, Kumamoto, Japan.; 3 Division of Carcinogenesis, The Cancer Institute, Japanese Foundation for Cancer Research, Tokyo, Japan.; 4 Department of Obstetrics and Gynecology, Graduate School of Medical Sciences, Kumamoto University, Kumamoto, Japan.; 5 Cancer Center, Promotion Headquarters, Fujita Health University, Aichi, Japan.; 6 Center for Metabolic Regulation of Healthy Aging, Faculty of Life Sciences, Kumamoto University, Kumamoto, Japan.

**Keywords:** Metabolism, Oncology, Cancer

## Abstract

Glycolysis is highly enhanced in pancreatic ductal adenocarcinoma (PDAC) cells; thus, glucose restrictions are imposed on nontumor cells in the PDAC tumor microenvironment (TME). However, little is known about how such glucose competition alters metabolism and confers phenotypic changes in stromal cells in the TME. Here, we report that cancer-associated fibroblasts (CAFs) with restricted glucose availability utilize lactate from glycolysis-enhanced cancer cells as a fuel and exert immunosuppressive activity in the PDAC TME. The expression of lactate dehydrogenase A (LDHA), which regulates lactate production, was a poor prognostic factor for patients with PDAC, and LDHA depletion suppressed tumor growth in a CAF-rich murine PDAC model. Coculture of CAFs with PDAC cells revealed that most of the glucose was taken up by the tumor cells and that CAFs consumed lactate via monocarboxylate transporter 1 to enhance proliferation through the TCA cycle. Moreover, lactate-stimulated CAFs upregulated IL-6 expression and suppressed cytotoxic immune cell activity synergistically with lactate. Finally, the LDHA inhibitor FX11 reduced tumor growth and improved antitumor immunity in CAF-rich PDAC tumors. Our study provides insight regarding the crosstalk among tumor cells, CAFs, and immune cells mediated by lactate and offers therapeutic strategies for targeting LDHA enzymatic activity in PDAC cells.

## Graphical abstract

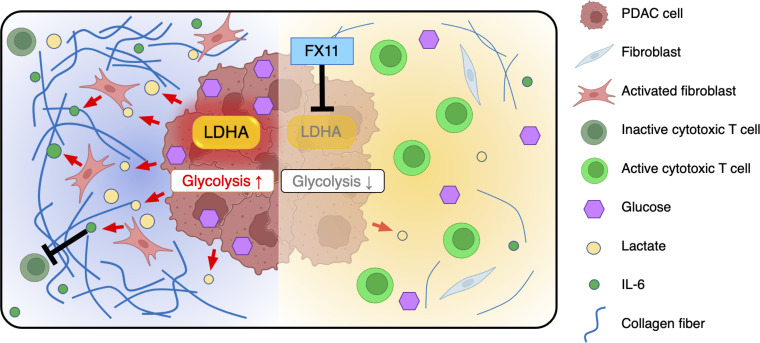

## Introduction

Pancreatic cancer is one of the most lethal types of cancer, and approximately 90% of pancreatic cancers are pancreatic ductal adenocarcinoma (PDAC). The incidence of PDAC is increasing annually, and PDAC is projected to have the second highest cancer-related mortality rate in 2030 (1). Currently, the treatment options for advanced PDAC are limited, and the development of more effective therapeutic strategies is urgently needed. Glucose uptake (2) and glycolysis (3) in PDAC cells are enhanced (Warburg effect) due to hypovascularity (4). Such deregulated glycolysis has been considered to have pivotal roles in tumor growth and progression by meeting the energetic demands of cancer cells that proliferate under oncogenic stimulation. Moreover, altered metabolites from cancer cells produce phenotypic changes in not only cancer cells themselves but also nontumor cells in the tumor microenvironment (TME). Thus, disrupting this dependency on glucose metabolism could be a promising strategy for PDAC treatment.

Most PDAC tumors are driven by aberrant KRAS activity (5), and a line of evidence suggests that oncogenic KRAS signaling enhances glycolysis (6) in cancer cells by increasing the expression of glycolytic enzymes, such as lactate dehydrogenase A (LDHA) (7). LDHA is a member of the lactate dehydrogenase (LDH) family, which includes central players in glycolysis and is known to be involved in the development and progression of cancer through lactate production (8). Lactate is produced during anaerobic glycolysis and was once thought to be a metabolic waste product. Lactate is now recognized to play crucial roles in vivo, including as an energy source for various types of cells and a mediator of intercellular communication (9), and studies have shown that lactate is also exploited by cancer cells to foster a tumor-supporting microenvironment, such as an immunosuppressive TME (10). For example, the accumulation of lactate derived from cancer cells in the TME has been shown to impair the cytokine production and proliferation of cytotoxic T lymphocytes, a central player in antitumor immunity, through lactate efflux blockade and disruption of T cell metabolism (11). In addition, a very recent study demonstrated that in a highly glycolytic TME, lactate eliminated the efficacy of an immune checkpoint inhibitor by modulating the balance of programmed cell death 1 (PD-1) expression between CD8^+^ T cells and regulatory T cells (12). Furthermore, lactate has a promotive effect on the infiltration of myeloid-derived suppressor cells into pancreatic tumors while decreasing the cytotoxic activity of natural killer (NK) cells (13).

On the other hand, many studies have shown the pivotal roles of cancer-associated fibroblasts (CAFs) in tumor progression, including promoting tumor cell growth and migration and suppressing antitumor immunity (14), and a recent study revealed that lactate conferred phenotypic features of mesenchymal stem cells to CAFs to promote tumor cell proliferation and invasion (15). However, little is known about whether and how tumor cell–derived lactate is involved in the formation of the CAF-rich TME and how it affects the immunosuppressive effects of CAFs. Here, we showed that CAFs in the glucose-starved PDAC TME utilized lactate from LDHA-active glycolytic tumor cells as an energy source to support proliferation and interleukin-6 (IL-6) production, which suppressed antitumor immune cells and accelerated PDAC tumor progression.

## Results

### LDHA enhances glycolysis and lactate production in PDAC cells.

**Figure 1:**
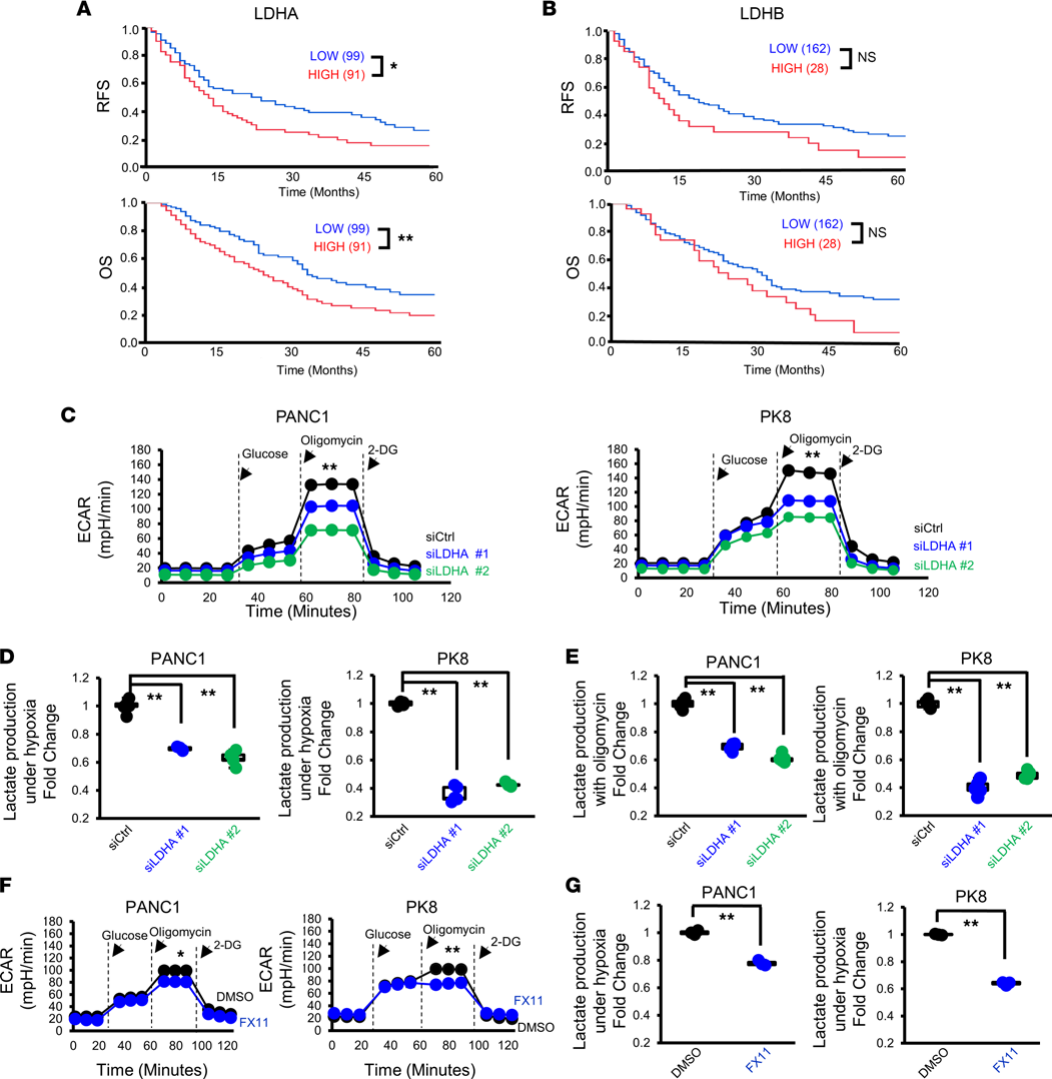
LDHA in PDAC cells regulates lactate production. (**A** and **B**) Kaplan-Meier survival analysis of the RFS (top) and OS (bottom) of patients with PDAC (*n* = 190) according to the LDHA (**A**) and LDHB (**B**) immunostaining indexes. The log-rank test was used to calculate *P* values. (**C**) Analysis of the glycolytic abilities of PANC-1 and PK8 cells using a flux analyzer after transfection with siCtrl or siLDHA (*n* = 3). (**D**) Measurement of the lactate concentration in the culture supernatants of PANC-1 and PK8 cells after transfection with siCtrl or siLDHA under hypoxic conditions (*n* = 3). (**E**) Measurement of the lactate concentration in the culture supernatants after administration of oligomycin to PANC-1 and PK8 cells transfected with siCtrl or siLDHA (*n* = 5). (**F**) Analysis of the glycolytic abilities of PANC-1 and PK8 cells treated with FX11 (*n* = 3). (**G**) The lactate concentration in the culture supernatants after administration of oligomycin to PANC-1 and PK8 cells treated with FX11. *P < 0.05; **P < 0.01. A Student’s *t* test was used to compare continuous variables between 2 groups. One-way ANOVA followed by Tukey’s multiple-comparison test was used to compare multiple groups. 2-DG, 2-deoxy-d-glucose.

We first investigated whether the expression of LDH family molecules, which are involved in lactate production, impacts the prognosis of patients with PDAC. We evaluated by immunohistochemistry the expression of LDHA and lactate dehydrogenase B (LDHB) in 190 resected PDAC tumor tissues from patients with clinical outcome information (Supplemental Figure 1, A–C; supplemental material available online with this article; https://doi.org/10.1172/jci.insight.163022DS1). We observed that LDHA-high patients (*n* = 91) exhibited poorer relapse-free survival (RFS) and overall survival (OS) than LDHA-low or LDHA-negative patients (*n* = 99) (Figure 1A). On the other hand, although we found trends connecting high LDHB expression with short RFS and OS, the differences were not statistically significant (Figure 1B). These results imply potential roles for LDHA in PDAC tumor progression, and we therefore focused on studying LDHA, not LDHB.

Given that LDHA plays a crucial role in regulating glycolysis, we performed cell metabolic analysis using an extracellular flux analyzer (Figure 1C) to assess the impact of LDHA inhibition on glycolysis in PDAC cells. We tested several PDAC cell lines (Supplemental Table 1) and selected highly lactate-producing PK8 cells and low-lactate-producing PANC-1 cells (Supplemental Figure 1D) for further experiments. The origins of these cells (i.e., PANC-1 is derived from the primary PDAC tumor while PK8 is established from the liver metastatic site) might be related to the difference in their lactate production. We observed that LDHA suppression by the siRNAs (Supplemental Figure 1E) significantly decreased the peak extracellular acidification rate (ECAR) after oligomycin treatment (Figure 1C), which indicates the maximum glycolytic capacity to convert glucose to lactate. Consistently, although there was no change under normoxic conditions (data not shown), the amount of lactate in the culture supernatant of LDHA-depleted PDAC cells was decreased under hypoxic conditions (Figure 1D). We also observed that lactate production in LDHA-depleted PDAC cells was decreased by oligomycin treatment (Figure 1E). Similarly, pharmacological inhibition of LDHA with FX11, a selective LDHA inhibitor (16, 17), significantly decreased the peak ECAR (Figure 1F) and lactate production (Figure 1G) after oligomycin treatment in both PDAC cell lines. LDHA has been reported to promote tumor cell growth in the past (18, 19). However, suppression of LDHA by siRNAs did not change the proliferative capacity of PANC-1 or PK8 human PDAC cells (Supplemental Figure 1F). Based on these results, we verified that LDHA regulates the glycolytic capacity and lactate production of PDAC cells.

### LDHA depletion in cancer cells reduces CAF levels in the PDAC TME.

**Figure 2:**
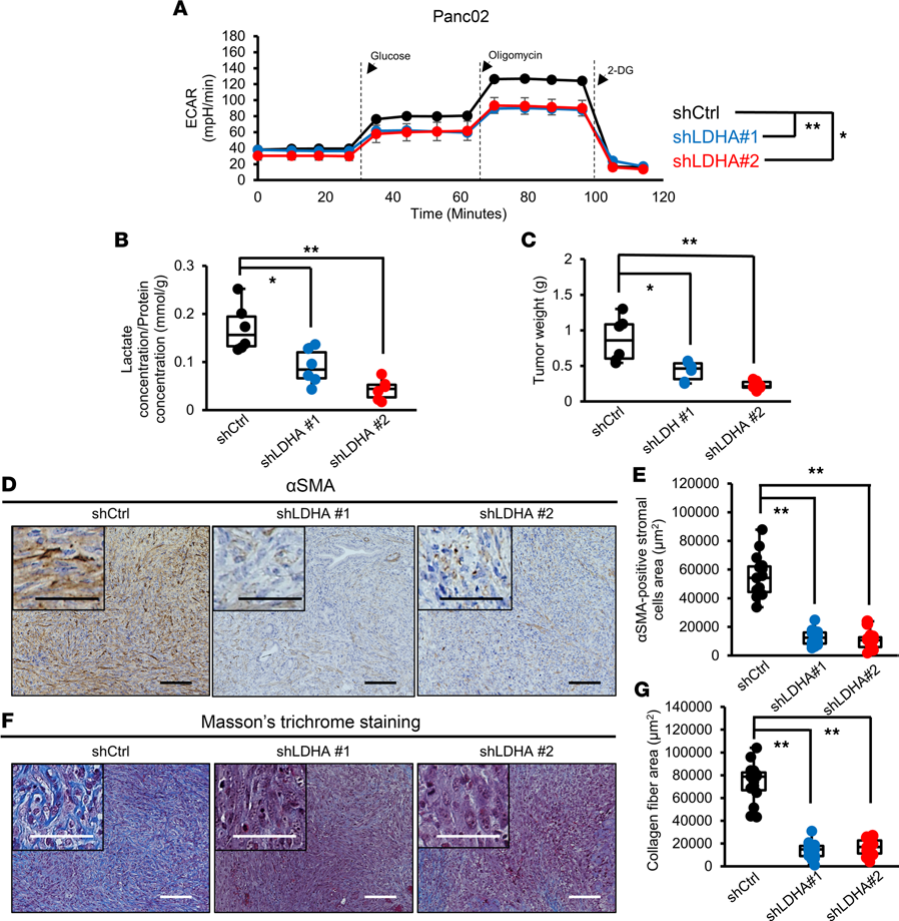
CAF numbers in the PDAC TME are reduced by LDHA KD in tumor cells. (**A**) Analysis of the glycolytic ability of Panc02 cells using a flux analyzer after knockdown of LDHA (*n* = 3). (**B**) Measurement of the lactate concentration in tumors from the Ctrl and LDHA-KD groups (*n* = 6). (**C**) Weight of tumors from the Ctrl and LDHA-KD groups (*n* = 6). (**D**) Representative images of αSMA immunohistochemical staining in resected tumors from the Ctrl and LDHA-KD groups. (**E**) Quantification of the αSMA-positive stromal area in Ctrl and LDHA-KD tumors (*n* = 15). (**F**) Representative images of Masson’s trichrome staining in tumors from the Ctrl and LDHA-KD groups. (**G**) Quantification of the blue-stained collagen fibers in the stromal area in Ctrl and LDHA-KD tumors (*n* = 15). Scale bars: 100 μm. Box plots show the interquartile range (box), median (line), and minimum and maximum (whiskers). *P < 0.05; **P < 0.01. A Student’s *t* test was used to compare continuous variables between 2 groups. One-way ANOVA followed by Tukey’s multiple-comparison test was used to compare multiple groups.

We next tested the impact of LDHA depletion on Panc02 murine PDAC cells. Similar to the results for the human PDAC cell lines, LDHA gene knockdown (KD) (Supplemental Figure 2A) significantly reduced the maximum glycolytic capacity of Panc02 cells (Figure 2A), and overexpression of the shRNA-resistant LDHA gene successfully rescued glycolytic activity (Supplemental Figure 2B). The proliferation of Panc02 cells was not affected by LDHA KD (Supplemental Figure 2C). Next, we inoculated control (shCtrl) or LDHA-KD Panc02 (shLDHA) cell lines into the pancreas of mice to generate orthotopic transplantation mouse models and analyzed tumors 3 weeks after cell inoculation. We found that the amount of lactate in tumors generated by shLDHA Panc02 cells was decreased compared with that in tumors generated by shCtrl Panc02 cells (shCtrl, Figure 2B). In addition, the tumor weights in the shLDHA groups were significantly smaller than those in the shCtrl group (Figure 2C).

A line of evidence suggests that the interaction between the TME and tumor cells mediated by lactate can facilitate the reprogramming of stromal cells and promote the proliferation of CAFs (15, 20–22), which play crucial roles in tumor progression (14). We found that the number of αSMA-positive CAFs was significantly reduced in the tumors of the shLDHA groups compared with that in the shCtrl group (Figure 2, D and E). We also verified that the deposition of collagen fibers was decreased by LDHA KD (Figure 2, F and G). These results imply that lactate derived from LDHA-expressing tumor cells plays a pivotal role in CAF proliferation, which leads to fibrotic tumor formation.

### CAFs are in a glucose-starved state in the PDAC TME.

**Figure 3:**
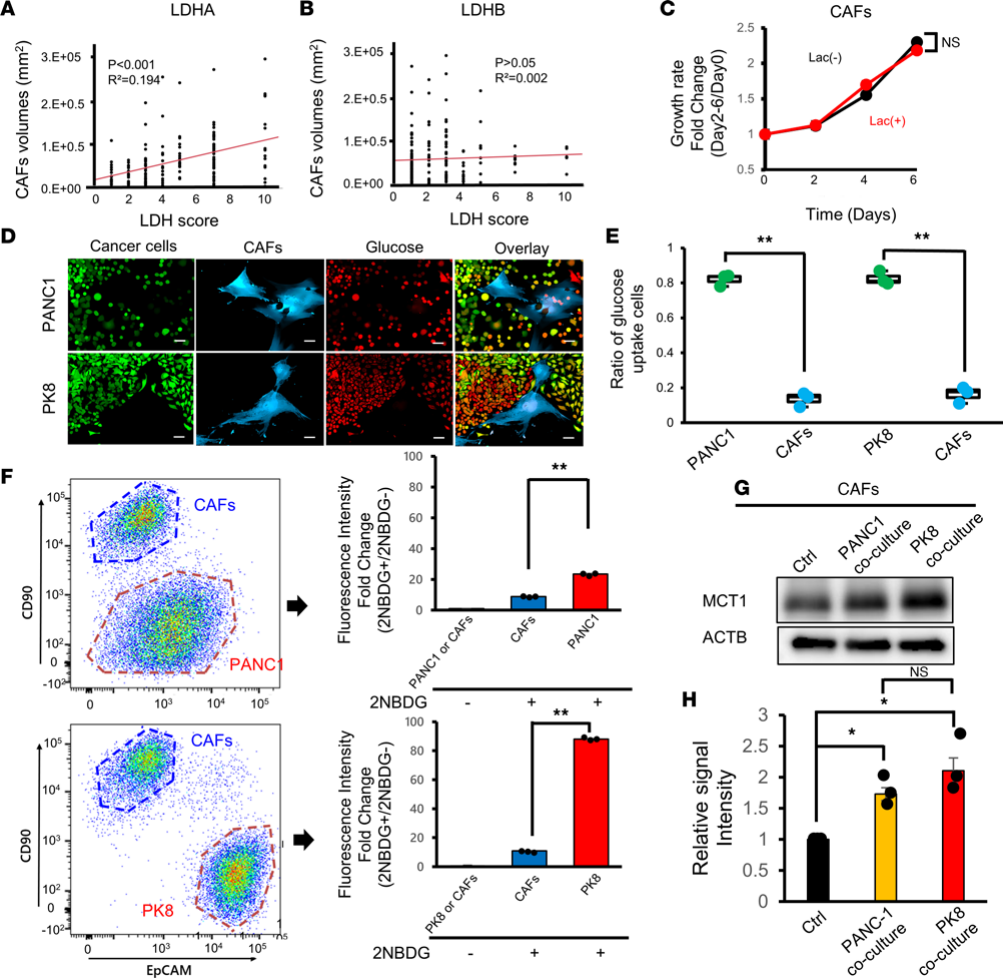
Metabolic competition between PDAC tumor cells and CAFs. (**A** and **B**) Correlations between the number of CAFs and the expression of LDHA (**A**) or LDHB (**B**) in PDAC patients (*n* = 190). (**C**) Growth assay performed with human CAFs stimulated with lactate in complete medium (*n* = 3). (**D**) Representative fluorescence microscopy images of PANC-1 (top) or PK8 (bottom) cells cultured with human CAFs and fluorescently labeled glucose. Scale bars: 50 μm. (**E**) Quantification of glucose uptake by PDAC cells or CAFs counted under a fluorescence microscope (*n* = 3). (**F**) Quantification of glucose uptake by PDAC cells or CAFs by flow cytometry. (**G**) Western blotting analysis of MCT1 expression in CAFs cocultured with PANC-1 or PK8 cells. (**H**) Quantification of the intensity of MCT1 in Western blot analysis in **G** (*n* = 3). *P < 0.05; **P < 0.01. A Student’s *t* test was used to compare continuous variables between 2 groups. One-way ANOVA followed by Tukey’s multiple-comparison test was used to compare multiple groups. 2NBDG, 2-[*N*-(7-nitrobenz-2-oxa-1,3-diazol-4-yl)amino]-2-deoxy-d-glucose.

Given the significance of LDHA expression in fibrotic tumor formation observed in the orthotopic mouse model, we next evaluated the CAF volume in 190 resected PDAC tumor tissues by αSMA staining (Supplemental Figure 3A) to investigate the relationship between CAFs and LDHA expression in human PDAC tumors. We showed that patients with many αSMA-positive CAFs had a significantly worse prognosis (Supplemental Figure 3B). We also found that the number of CAFs was positively correlated with LDHA expression (Figure 3A) but not with LDHB expression (Figure 3B). These results further suggest that lactate from LDHA-active PDAC tumor cells increases the number of CAFs, which promote fibrotic tumor formation.

To test the effect of lactate on the growth of CAFs, we isolated CAFs from PDAC patient tumor tissues and treated them with lactate under normal culture conditions in RPMI 1640 medium supplemented with 10% fetal bovine serum (FBS). However, we observed no obvious effect of lactate on CAF proliferation (Figure 3C). Given that evidence suggests that cancer cells impose nutrient restrictions on nontumor cells in the TME (23, 24), we hypothesized that CAFs could use lactate as a fuel in the presence of tumor cells that monopolize glucose in the PDAC TME. To test this hypothesis, we performed glucose competition assays by directly coculturing CAFs with PDAC cells and adding fluorescently labeled glucose (Supplemental Figure 3C). Strikingly, we demonstrated that most of the fluorescently labeled glucose was taken up by the cancer cells, and the percentage of CAFs that took up glucose was significantly smaller than that of cancer cells by microscopic examination (Figure 3, D and E). Flow cytometric evaluation also revealed that compared with CAFs, cancer cells predominantly took up glucose in direct cocultures (Figure 3F). Since we observed that the glucose uptake of PK8 cells was much higher than that of PANC-1 cells (Figure 3F), we tested the expression of glucose transporter 1 (GLUT1), one of the major receptors for glucose (25), in PANC-1 and PK8 cells and found that GLUT1 expression was significantly higher in PK8 cells than in PANC-1 cells (Supplemental Figure 3D), accounting for the higher glucose uptake in PK8 cells. On the other hand, the expression of monocarboxylate transporter 1 (MCT1) in CAFs, a major transporter involved in the uptake of lactate (26, 27), was upregulated by coculturing with PANC-1 and PK8 cells (Figure 3G and Supplemental Figure 3E), suggesting that the uptake of lactate by CAFs is increased through MCT1 induction. Furthermore, we found that MCT1 expression in CAFs was more increased when they were cocultured with highly lactate-producing PK8 cells than when they were cocultured with low-lactate-producing PANC-1 cells (Figure 3H). In addition, knockdown of the LDHA gene in PDAC cells decreased MCT1 expression in cocultured CAFs (Supplemental Figure 3F). These positive correlations between MCT1 expression in CAFs and LDHA expression in PDAC cells in the coculture assay suggest that lactate uptake in CAFs can be increased in glycolytic tumors by upregulation of MCT1 expression in CAFs in the PDAC TME.

### CAFs utilize lactate as an energy source through the TCA cycle.

**Figure 4:**
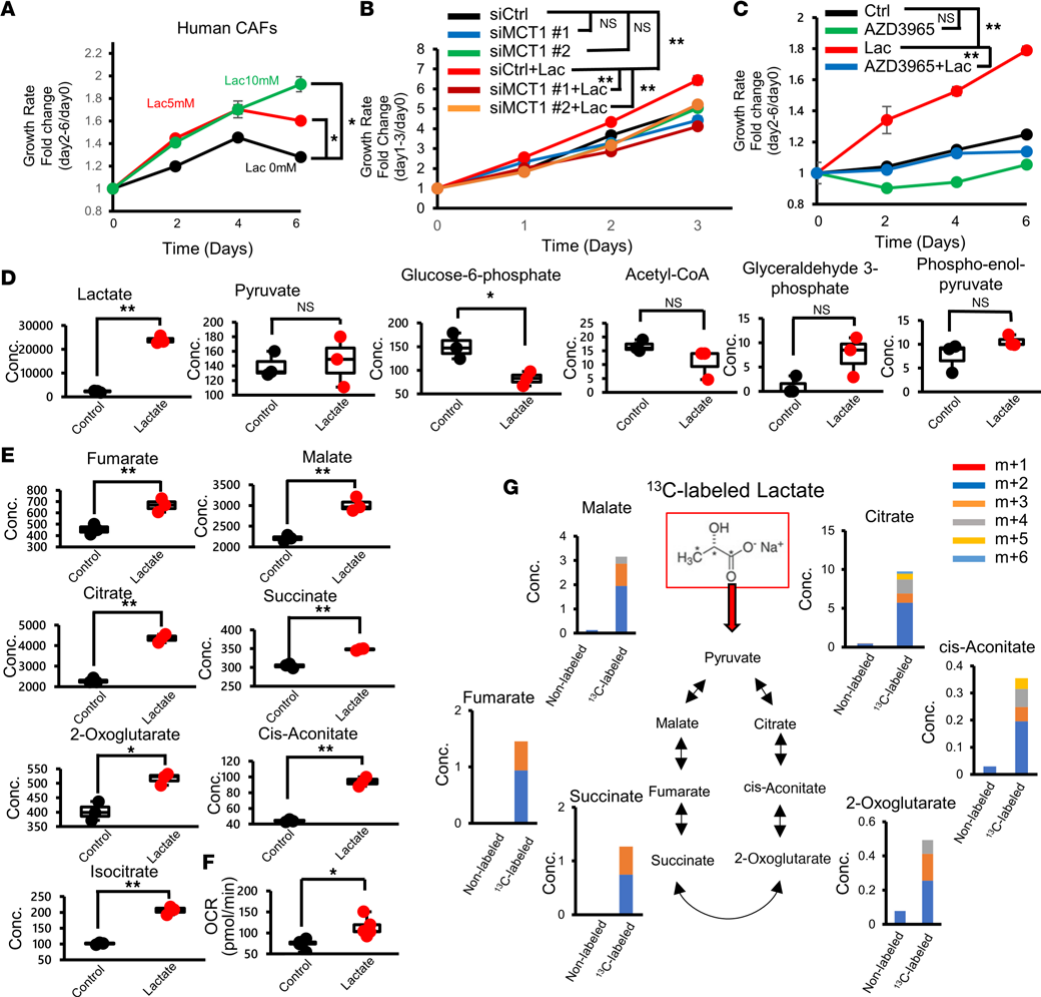
CAFs use lactate as a fuel via the TCA cycle. (**A**) Growth assay performed with human CAFs with or without lactate in glucose-free medium (*n* = 3). (**B**) Growth curve of MCT1-KD CAFs stimulated with lactate (*n* = 3). (**C**) Growth assay performed with human CAFs with or without lactate ± AZD3965 in glucose-free medium (*n* = 3). (**D**) Comprehensive metabolite analysis of CAFs in the presence of lactate in glucose metabolism (*n* = 3). (**E**) Comprehensive metabolite analysis of CAFs in the presence of lactate in the TCA cycle (*n* = 3). (**F**) Oxygen consumption rate (OCR) of human CAFs after 24 hours of lactate stimulation (*n* = 5). Box plots show the interquartile range (box), median (line), and minimum and maximum (whiskers). (**G**) Isotopologue distribution of metabolites associated with the TCA cycle in CAFs treated with ^13^C-labeled or unlabeled lactate. *P < 0.05; **P < 0.01. A Student’s *t* test was used to compare continuous variables between 2 groups. One-way ANOVA followed by Tukey’s multiple-comparison test was used to compare multiple groups.

To test whether lactate stimulates the proliferation of CAFs in a glucose-starved state, we treated CAFs with medium containing lactate but not glucose. As expected, CAF growth was enhanced by lactate treatment (Figure 4A and Supplemental Figure 4A), while adding lactate did not enhance the proliferation of PDAC cells cultured with glucose-free medium (Supplemental Figure 4B). Notably, we showed that siRNA-mediated MCT1 gene suppression (Supplemental Figure 4C) or AZD3965 (26, 28), an inhibitor of MCT1, eliminated the effect of lactate on human CAF proliferation under glucose-starved conditions (Figure 4, B and C). Furthermore, we observed that conditioned medium derived from PANC-1 or PK8 cells stimulated CAF growth, and MCT1 KD in CAFs abolished these effects (Supplemental Figure 4D). These results suggest that lactate could be a nutrient for glucose-deprived CAFs in the PDAC TME.

To further investigate how CAFs metabolize lactate, a comprehensive metabolomic analysis of lactate-loaded CAFs was performed. Lactate administration markedly increased the lactate concentration in CAFs but did not increase the levels of metabolites upstream of glucose metabolism (Figure 4D). On the other hand, the levels of metabolites involved in the tricarboxylic acid (TCA) cycle were elevated (Figure 4E and Supplemental Figure 4E). Moreover, an extracellular flux analyzer revealed that the oxygen consumption rate (OCR) of CAFs was increased after lactate exposure (Figure 4F). We further performed a ^13^C-labeled lactate isotope tracing experiment and observed increased ^13^C-labeled metabolites involved in the TCA cycle (Figure 4G), suggesting that administered ^13^C-labeled lactate was successfully metabolized in the TCA cycle as a carbon source. Taken together, these results suggest that CAFs consume lactate as an energy source via the TCA cycle to support proliferation in the low-glucose PDAC TME.

### Lactate stimulates CAFs to produce IL-6 to synergistically suppress antitumor immunity.

**Figure 5:**
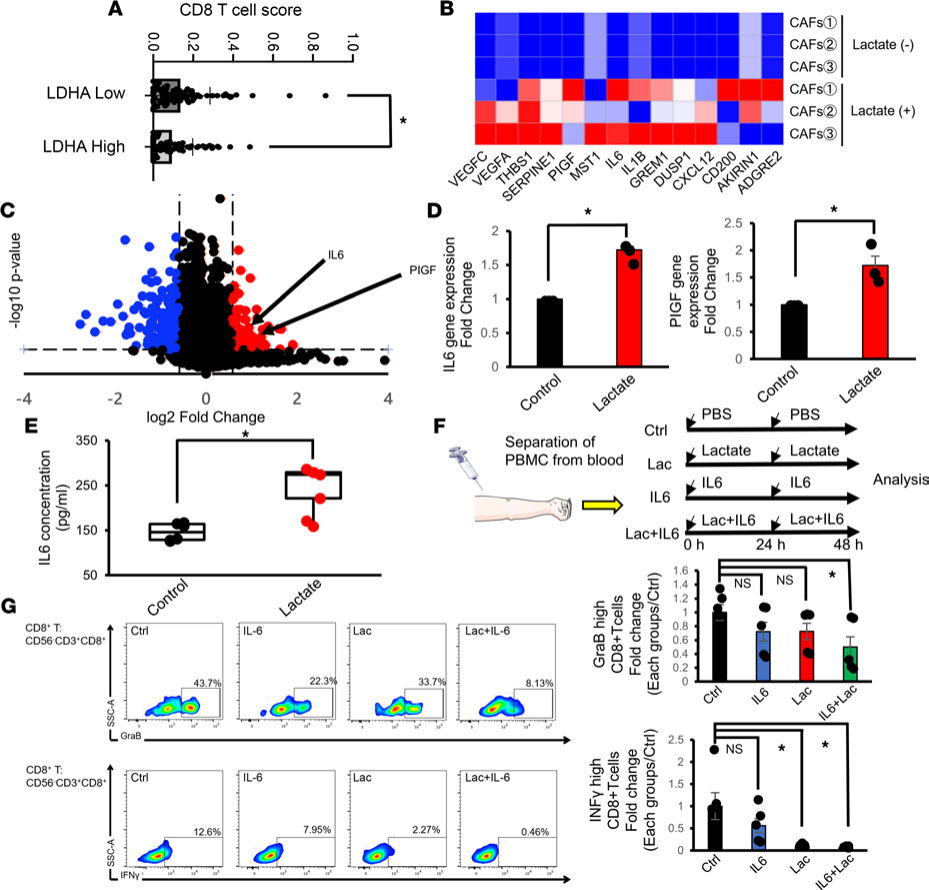
Lactate-stimulated CAFs produce IL-6 and suppress antitumor immunity. (**A**) CIBERSORT analysis of estimated CD8^+^ T cell infiltration in tumors in the LDHA-high and LDHA-low subgroups of PDAC patients from the TCGA cohort (*n* = 177). (**B**) Heatmap of the mRNA expression of immunosuppression-related genes in CAFs stimulated with or without lactate (*n* = 3). (**C**) Volcano plot of the mRNA expression of CAFs stimulated with or without lactate (*n* = 3). (**D**) Quantification of *IL6* and *PIGF* expression in CAFs stimulated with or without lactate by quantitative PCR (*n* = 3). (**E**) Quantification of the IL-6 concentration in the conditioned medium of CAFs stimulated with or without lactate (*n* = 6). Box plots show the interquartile range (box), median (line), and minimum and maximum (whiskers). (**F**) Schematic of the experimental model for analysis of the cytotoxic activity of CD8^+^ T cells. PBMCs were isolated from healthy donors and stimulated with PBS, lactate, IL-6, or lactate plus IL-6. After 48 hours, the cells were analyzed by flow cytometry. (**G**) Quantification of GraB (top) and IFN-γ (bottom) expression in CD8^+^ T cells stimulated as described in **F** by flow cytometry (*n* = 3). *P < 0.05. A Student’s *t* test was used to compare continuous variables between 2 groups. One-way ANOVA followed by Tukey’s multiple-comparison test was used to compare multiple groups.

Next, we investigated how lactate-stimulated CAFs are involved in fostering the tumor-promoting TME in PDAC. By analyzing RNA-sequencing data for PDAC patients in a data set from The Cancer Genome Atlas (TCGA) by CIBERSORT (29), a computational deconvolution algorithm used to predict the immune cell composition from bulk gene expression data, we found that PDAC patients with high *LDHA* gene expression had fewer CD8^+^ T cells than those with low *LDHA* gene expression (Figure 5A), consistent with reports demonstrating that lactate inhibits T cell motility and that LDHA-associated lactate reduces the infiltration of T cells into tumors (30, 31). To elucidate the roles of CAFs in lactate-mediated immunosuppression, we compared the gene expression profiles of CAFs with or without lactate stimulation by bulk RNA sequencing. We found that the expression of genes related to immunosuppression was significantly upregulated in lactate-stimulated CAFs (Figure 5B), and the expression of *IL6* and phosphatidylinositol glycan anchor biosynthesis class F (*PIGF*) was significantly enhanced (Figure 5C). The enhanced expression of the *IL6* and *PIGF* genes in lactate-stimulated CAFs was further verified by quantitative PCR (Figure 5D and Supplemental Table 2). We also found that the concentration of IL-6 in CAF-conditioned medium was increased by lactate treatment (Figure 5E), while PIGF was not detected in conditioned medium from either control or lactate-stimulated CAFs (data not shown). Furthermore, we verified a significant decrease in the concentration of IL-6 in the conditioned medium from MCT1-KD CAFs stimulated with lactate (Supplemental Figure 5A), suggesting the crucial role of lactate in IL-6 production in CAFs.

We next tested whether there is a synergistic suppressive effect on the cytotoxic function of lymphocytes induced by the combination of lactate and IL-6. Peripheral blood mononuclear cells (PBMCs) isolated from the blood of healthy volunteers were stimulated with lactate and recombinant IL-6 (Figure 5F). Although the decrease in granzyme B (GraB) and interferon-γ (IFN-γ) expression in CD8^+^ T cells in PBMCs was not remarkable when these cells were treated with lactate or IL-6 alone, the combination of lactate and IL-6 significantly reduced the expression of both GraB and IFN-γ in CD8^+^ T cells (Figure 5G). Moreover, GraB and IFN-γ expression in NK cells in PBMCs was decreased by lactate or IL-6 treatment, and the combined use of lactate and IL-6 resulted in further decreases in both GraB and IFN-γ (Supplemental Figure 5B). Together, these results suggest that lactate from cancer cells stimulates CAFs to produce IL-6, synergistically impairing the functions of cytotoxic lymphocytes.

### Pharmacological inhibition of LDHA decreases lactate production in PDAC cells, reduces CAFs, and improves antitumor immunity in a subcutaneous PDAC mouse model.

**Figure 6:**
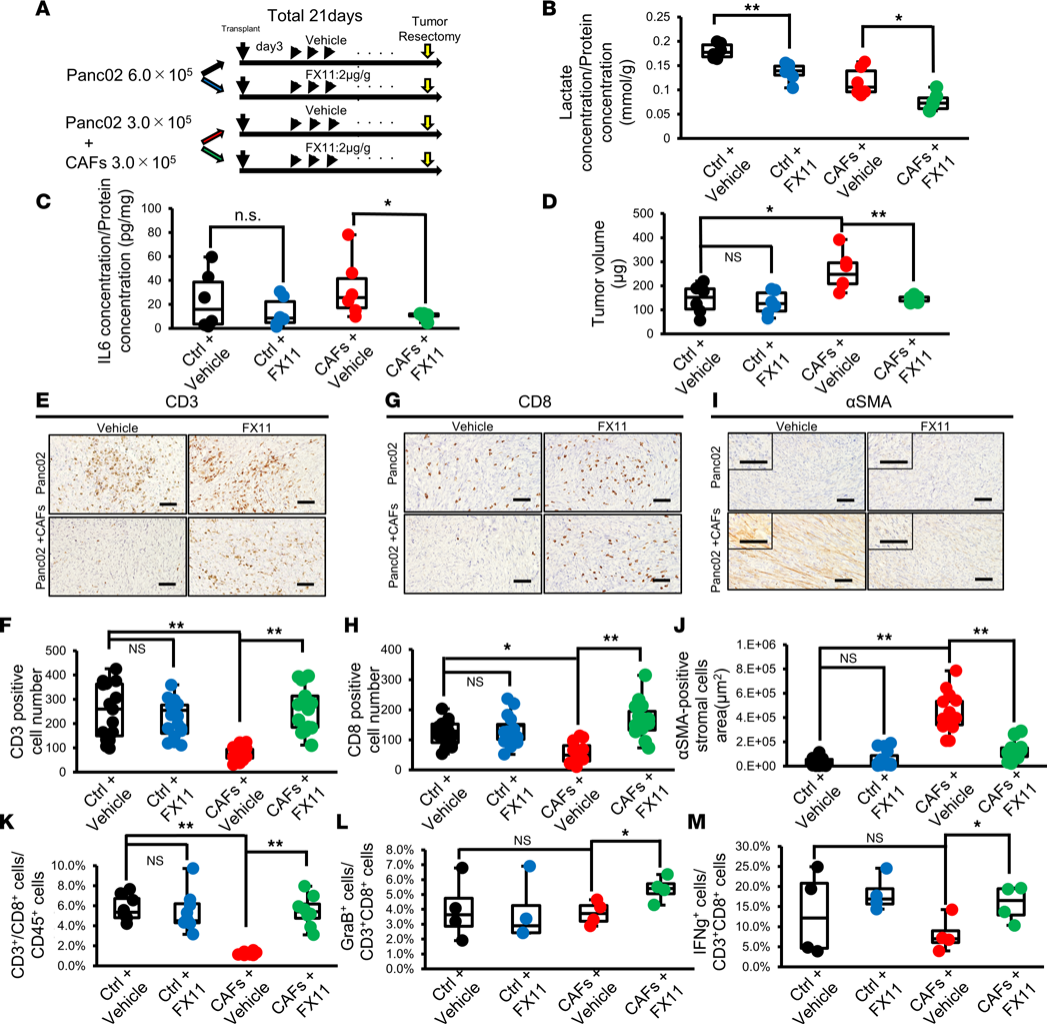
Pharmacological inhibition of LDHA decreases lactate production in PDAC cells, reduces CAFs, and improves antitumor immunity in a subcutaneous PDAC mouse model. (**A**) Schematic representation of the experimental protocol. Panc02 cells with or without murine CAFs were inoculated into C57BL/6 mice. After 3 days, the mice were treated daily with vehicle control or FX11 until day 20. The mice were sacrificed on day 21. (**B**) Measurement of the lactate concentration in tumors from the Ctrl or CAF group treated with vehicle or FX11 (*n* = 6). (**C**) Measurement of the IL-6 concentration in tumors from the Ctrl or CAF group treated with vehicle or FX11 (*n* = 6). (**D**) Weight of tumors from the Ctrl or CAF group harvested at 21 days after transplantation (*n* = 6). (**E**–**J**) Representative images for CD3 (**E**), CD8 (**G**), or αSMA (**I**) immunohistochemical staining and quantification of the number of CD3^+^ (**F**) or CD8^+^ (**H**) lymphocytes or αSMA-positive cells (**J**) in resected tumors from the Ctrl or CAF group treated with vehicle control or FX11. Scale bars: 100 μm. (**K**–**M**) Flow cytometric analysis for quantification of the percentages of CD3^+^CD8^+^ T cells in CD45^+^ cells (**K**), GraB^+^ cells in CD3^+^CD8^+^ T cells (**L**), and IFN-γ^+^ cells in CD3^+^CD8^+^ T cells (**M**) infiltrated into tumors from the Ctrl or CAF group treated with vehicle control or FX11 (*n* = 8). Box plots show the interquartile range (box), median (line), and minimum and maximum (whiskers). *P < 0.05; **P < 0.01. A Student’s *t* test was used to compare continuous variables between 2 groups. One-way ANOVA followed by Tukey’s multiple-comparison test was used to compare multiple groups.

In addition to that of genetic depletion of LDHA, the therapeutic effect of pharmacological inhibition of LDHA by FX11 was investigated in subcutaneous tumors generated by Panc02 cells alone (Ctrl group) or Panc02 cells implanted with CAFs (CAF group), which enhance fibrotic stroma (Figure 6A). FX11 treatment decreased the peak ECAR of Panc02 cells in vitro (Supplemental Figure 6A) and the lactate concentration in the tumors in the Ctrl and CAF groups (Figure 6B). The body weight of mice was not decreased by FX11 (Supplemental Figure 6B). Importantly, FX11 treatment significantly suppressed the IL-6 concentration in the tumors in the CAF group, suggesting that CAFs are a major source of IL-6 (Figure 6C). Moreover, in the Ctrl group, there was no difference in tumor weight between vehicle and FX11 treatment, whereas administration of FX11 significantly reduced tumor weight in the CAF group (Figure 6D and Supplemental Figure 6, C and D). Immunohistochemical staining revealed that the infiltration of CD3^+^ and CD8^+^ lymphocytes was decreased in the CAF group compared with the Ctrl group and that lymphocyte infiltration was increased by the administration of FX11 (Figure 6, E–H). We also verified that the number of αSMA-positive CAFs was significantly decreased by FX11 treatment in the CAF group (Figure 6, I and J). Moreover, flow cytometric analysis demonstrated that the number of CD8^+^ T lymphocytes in tumors in the CAF group was reduced compared with that in tumors in the Ctrl group (Figure 6K). Finally, in the tumors in the CAF groups, FX11 treatment increased the tumor infiltration of CD8^+^ T cells and NK cells (Figure 6K and Supplemental Figure 6E) as well as the expression of GraB and IFN-γ in CD8^+^ T cells in the tumors (Figure 6, L and M). Thus, pharmacological inhibition of LDHA suppressed tumor growth along with CAF reduction and reversed the immunosuppressive status of CAF-rich PDAC tumors through reductions in the levels of lactate and IL-6.

### LDHA inhibition ameliorates the fibrotic and immunosuppressive TME in orthotopic PDAC mouse models.

**Figure 7:**
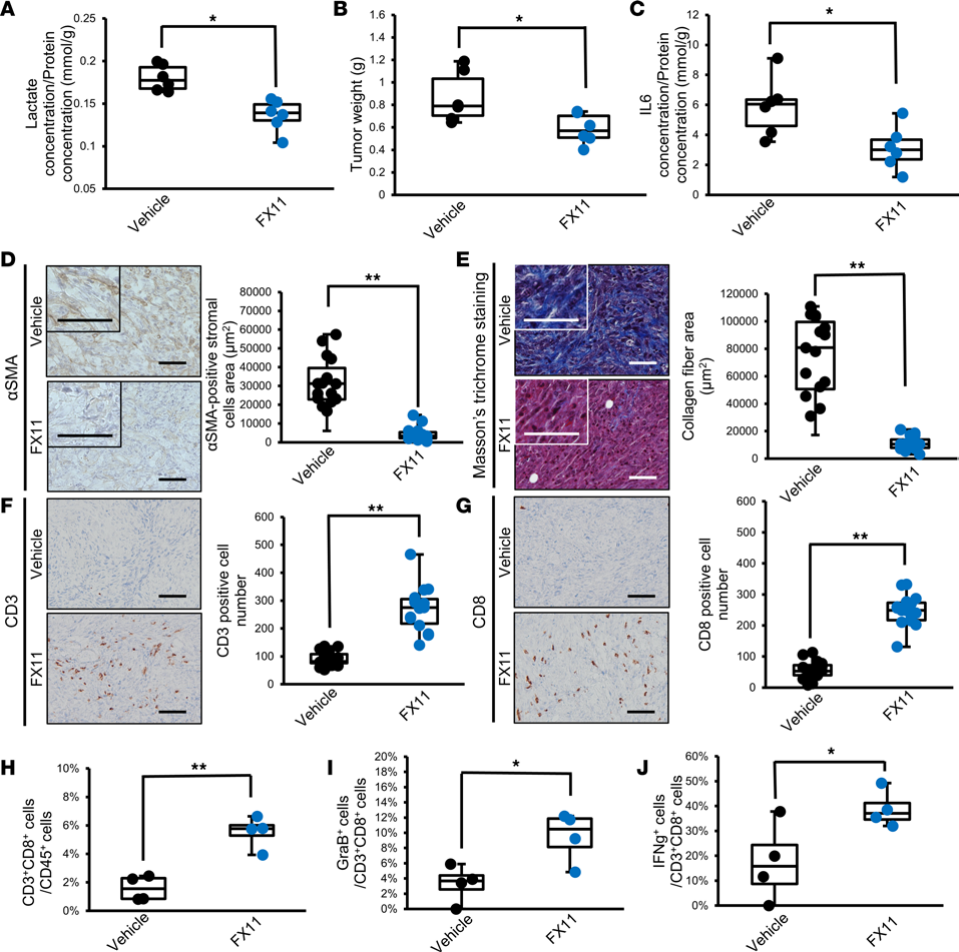
LDHA inhibition ameliorates the fibrotic and immunosuppressive TME in orthotopic PDAC mouse models. (**A**) Measurement of the lactate concentration in tumors from the vehicle- or FX11-treated group (*n* = 6). (**B**) Weights of tumors from the vehicle- or FX11-treated groups (*n* = 6). (**C**) The IL-6 concentration in tumor lysates from vehicle- or FX11-treated mice (*n* = 6). (**D**) Representative images of αSMA immunohistochemical staining and quantification of the αSMA-positive cell area in the vehicle or FX11 group (*n* = 15). (**E**) Representative images of Masson’s trichrome staining and quantification of the blue-stained collagen fiber area in the vehicle or FX11 group (*n* = 15). (**F** and **G**) Representative images of CD3 (**F**) and CD8 (**G**) immunohistochemical staining and quantification of the number of CD3^+^ (**F**) or CD8^+^ (**G**) lymphocytes in tumors from the vehicle- or FX11-treated group (*n* = 15). Scale bars: 100 µm. (**H**–**J**) Flow cytometric analysis for quantification of the percentages of CD3^+^CD8^+^ T cells in CD45^+^ cells (**H**), GraB^+^ cells in CD3^+^CD8^+^ T cells (**I**), and IFN-γ^+^ cells in CD3^+^CD8^+^ T cells (**J**) infiltrated into tumors treated with vehicle control or FX11 (*n* = 4). Box plots show the interquartile range (box), median (line), and minimum and maximum (whiskers). *P < 0.05; **P < 0.01. A Student’s *t* test was used to compare continuous variables between 2 groups. One-way ANOVA followed by Tukey’s multiple-comparison test was used to compare multiple groups.

Next, to test the effect of LDHA inhibition by FX11 or LDHA KD on a more clinically relevant model, we used an orthotopic Panc02 mouse model that recapitulates the PDAC TME. We observed that the lactate concentration in tumors (Figure 7A), tumor weight (Figure 7B), and IL-6 concentration in tumors (Figure 7C) were significantly decreased by FX11 treatment. We also verified that LDHA KD strongly reduced the IL-6 concentration in tumors (Supplemental Figure 7A). We also found that the number of αSMA-positive CAFs and collagen fiber deposition were decreased by FX11 (Figure 7, D and E), consistent with the results of shLDHA Panc02 tumors (Figure 2, D–G). Moreover, histological analyses revealed that the infiltration of CD3^+^ and CD8^+^ lymphocytes was increased by FX11 treatment (Figure 7, F and G) and genetic silencing of *LDHA* (Supplemental Figure 7, B and C). In addition, the tumor infiltration of CD8^+^ T cells and the expression of GraB and IFN-γ were increased by FX11 (Figure 7, H–J), and similar trends in the expression of GraB and IFN-γ were also observed in tumors with LDHA silencing (Supplemental Figure 7, D and E). These results further support that LDHA inhibition ameliorates the fibrotic and immunosuppressive pancreatic TME.

## Discussion

Enhanced glycolysis in cancer cells, known as the Warburg effect, is a strategic metabolic adaptation by tumors to satisfy their increased requirements for energy to support continuous proliferation and leads to the development of a tumor-supportive microenvironment by altering metabolites (32, 33). LDHA, which catalyzes pyruvate into lactate, is a central player in glycolysis, and it has been reported that LDHA is involved in tumor progression through lactate production in many types of cancer (17, 34, 35). Consistent with previous reports (36, 37), our clinical cohort revealed that LDHA expression in tumors was strongly correlated with a poor prognosis in PDAC patients, suggesting crucial roles for LDHA and lactate in PDAC progression. Given that lactate is involved in cancer progression (38) by affecting the TME, such as by promoting angiogenesis (39, 40) and suppressing antitumor immunity (31, 41), we sought to elucidate the tumor-promoting roles of lactate in the PDAC TME.

CAFs are the predominant component of the TME and have been extensively reported to support tumor progression (42–44) through various mechanisms, such as drug resistance (45) and immunosuppression (46). Recent evidence suggests that crosstalk between CAFs and tumor cells is mediated by lactate, which promotes cancer progression (9, 21). For instance, lactate derived from CAFs with enhanced glycolytic activity fueled breast cancer cell growth (47), and CAF-secreted lactate induced lipid metabolic reprogramming that enhanced the invasive capacity of prostate cancer (48). On the other hand, the accumulated lactate in the TME derived from tumor cells has also been suggested to be utilized by CAFs. In colorectal tumors, immunohistochemical analyses showed a high expression of MCT1 and a low level of GLUT1 expression in CAFs, while cancer cells exhibited strong expression of GLUT1, suggesting that CAFs take up tumor cell–derived lactate (20). In the current study, we established a direct coculture assay using PDAC patient–derived CAFs and human PDAC cell lines to demonstrate that nutrient restriction was imposed on CAFs in the TME due to exclusive glucose consumption by PDAC cells and that CAFs took up lactate instead via increased MCT1 expression. Consistent with our findings, prostate cancer cell–derived lactate can be taken up by the myofibroblast line WPMY-1 via the TCA cycle (49). In addition, we verified that PDAC patient–derived CAFs utilized lactate to promote growth through the TCA cycle under glucose-deprived conditions, underscoring lactate as the energy source for CAFs to promote the fibrotic milieu in the nutrient-limited PDAC TME. In addition, we identified that lactate stimulated CAFs to secrete various immunosuppressive cytokines, including IL-6 (50–52), through MCT1. Given that lactate is known to activate IL-6 regulatory pathways, such as nuclear factor-κB and mitogen-activated protein kinase, in many types of cells (53), further study is warranted to examine whether similar mechanisms contribute to increasing IL-6 production in lactate-stimulated CAFs.

A line of evidence has shown that metabolic competition in the TME is involved in the suppression of antitumor immunity. For example, highly glycolytic tumor cells outcompete T cells for glucose to suppress the cytotoxic function of T cells (23, 24). Moreover, lactate produced by tumor cells directly inhibits the antitumor activity of T cells by blocking lactate efflux and disrupting metabolism (11) and suppresses T cell infiltration into tumors (31). Our in vitro and in vivo experiments suggest that metabolic competition by glycolytic PDAC tumor cells leads to the accumulation of lactate in the TME and that CAFs utilize lactate to produce IL-6, which inhibits antitumor immunity. Because whole tumor lysates were used to measure the concentrations of lactate and IL-6 in the current study, cell type–specific measurements by purification of CAFs and tumor cells from tumors will further support the role of lactate-stimulated CAFs in fostering an immunosuppressive TME.

Although immune checkpoint blockade (ICB) therapy holds great promise for cancer treatment, effective ICB therapy for PDAC has not been established, and it is still necessary to define biomarkers that identify ICB responders and strategies to improve ICB efficacy. Accumulating evidence suggests the efficacy of the combination of a glycolysis inhibitor and ICB in tumors with enhanced glycolysis (12, 54). Our study revealed that the number of αSMA-positive CAFs was correlated with the expression of LDHA, a master regulator of glycolysis, in PDAC tumors and that PDAC patients with high LDHA mRNA expression had a low number of tumor-infiltrating CD8^+^ T cells, reflecting an immunosuppressed status. Our study also showed that pharmacological LDHA inhibition by FX11 successfully reduced tumor volumes and increased both the intratumoral infiltration and cytotoxic activity of CD8^+^ T cells in CAF-rich PDAC tumors. Therefore, the number of CAFs and the expression of LDHA could be biomarkers for predicting the efficacy of ICB therapies and glycolysis inhibitors. The PD-1/programmed cell death ligand 1 (PD-L1) axis is the most extensively investigated immune checkpoint. However, the response to immune checkpoint inhibitors targeting PD-1/PD-L1 is limited in PDAC, implying the possible involvement of different types of immune checkpoint molecules in the suppression of antitumor immunity. Indeed, a recent study demonstrated that CD155, a ligand for T cell immunoreceptor with immunoglobulin and ITIM domains (TIGIT), is highly expressed in human PDAC tumors, and combination therapy including an agonistic anti-CD40 antibody and coblockade of both PD-1 and TIGIT showed an enhanced effect on preclinical PDAC mouse models (55). Thus, further studies are warranted to determine which immune checkpoint molecules are predominantly involved in immunosuppression in CAF-rich, highly glycolytic PDAC tumors to develop effective ICB therapies administered in combination with glycolysis inhibitors.

**Figure 8:**
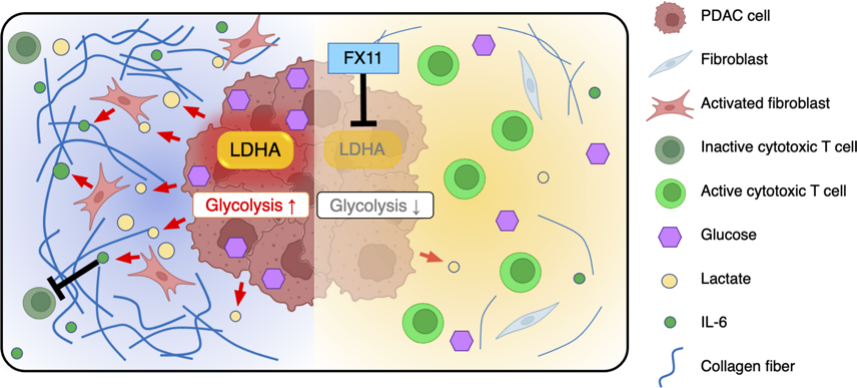
Schematic model. Lactate produced by LDHA-active PDAC cells promotes the proliferation of CAFs, which suppress antitumor immunity by secreting IL-6. Inhibition of LDHA by FX11 decreases lactate production in PDAC cells, leading to amelioration of immunosuppression in the TME.

In conclusion, we demonstrated that LDHA-active PDAC cells monopolize glucose and produce high amounts of lactate in the TME. Glucose-depleted CAFs take up lactate for use as an energy source and further produce IL-6, leading to the formation of an immunosuppressive TME in synergy with lactate and promoting tumor progression. Pharmacological inhibition of LDHA with FX11 improved the infiltration and antitumor activity of cytotoxic lymphocytes while reducing the number of CAFs in tumors and suppressing PDAC tumor growth by inhibiting the growth of CAFs and normalizing the immune response (Figure 8). Our study provides insight into the lactate-mediated crosstalk among tumor cells, CAFs, and immune cells in the TME and a rationale for the therapeutic strategy of targeting LDHA in glycolytic PDAC.

## Methods

### Patients and tissue samples.

Primary CAFs were isolated from patients with pancreatic cancer who underwent pancreatectomy without preoperative treatment at Kumamoto University after informed consent was obtained from each patient.

### Animal experiments.

Six- to seven-week-old male C57BL/6N mice (CLEA Japan) were housed in a room under stable temperature and humidity conditions on a 12-hour light/12-hour dark cycle. Both water and food were supplied ad libitum.

Panc02 cells (5.0 × 10^5^) were transplanted subcutaneously into mice, excised 4 weeks later, cultured in vitro, and used for animal experiments.

In the orthotopic mouse models, 6.0 × 10^5^ Panc02 cells, shCtrl Panc02 cells, or shLDHA Panc02 cells were orthotopically injected into the mouse pancreas. In the subcutaneous models, a mixture of Panc02 cells (3.0 × 10^5^) and mouse CAFs (3.0 × 10^5^) was subcutaneously injected. For LDHA inhibitor treatment experiments, FX11 (2.2 μg/g) or vehicle was intraperitoneally administered daily from day 3 to day 20.

Mouse body weight was evaluated twice per week. The mice in all experiments were sacrificed on day 21. Tumors were harvested from mice and subjected to further experiments.

### Cell lines and cell culture.

The human pancreatic cancer cell lines PK8 and PANC-1 were obtained from the Japanese Collection of Research Bioresources Cell Bank (Osaka, Japan) and the RIKEN Bio-Resource Center Cell Bank (Ibaraki, Japan). The mouse pancreatic cancer cell line Panc02 is available from ATCC. The murine cancer cell line (Panc02) was provided by T. Moroishi (Department of Cell Signaling and Metabolic Medicine, Faculty of Life Sciences, Kumamoto University). Human CAFs were established from surgically excised pancreatic cancer samples (56). Mouse CAFs, as described in the literature (57), were obtained by isolation of fibroblasts from mouse skin and stimulation of the cells with 2 ng/mL recombinant murine TGF-β (R&D Systems) for 6 days. These cells were cultured in RPMI 1640 medium or DMEM containing 10% FBS (normal medium) and incubated at 37°C with 5% CO_2_.

### Immunofluorescence and immunohistochemistry.

Immunohistochemistry was performed using 3 μm–thick specimens obtained from formalin-fixed, paraffin-embedded sections of pancreatic tumors from mice. We stained the sections for LDHA (Cell Signaling Technology), αSMA (Abcam), CD3 (Abcam), and CD8 (Abcam) (Supplemental Table 3) and selected the most invasive tumor area on each slide. Masson’s trichrome staining was performed on 2 μm–thick specimens. In brief, the sections were deparaffinized and rehydrated, and the LDHA antigen was activated by microwaving in a buffer solution (pH 6) for 15 minutes. The αSMA, CD3, and CD8 antigens were activated by autoclaving with a buffer solution (pH 9) for 15 minutes. The sections were incubated with the appropriate aforementioned antibody overnight at 4°C, followed by incubation with an anti-rabbit IgG HRP secondary antibody (DAKO) for 30 minutes at room temperature. The slides were developed with 3,3′-diaminobenzidine (DAKO) and counterstained with hematoxylin.

In Figure 1, A and B, the proportion of LDHA- or LDHB-positive cells in pancreatic tumor tissues was scored as follows: 1, 0%–33% positive tumor cells; 2, 34%–66% positive tumor cells; and 3, 67%–100% positive tumor cells. Staining intensity was scored according to the following criteria: 0, negative, no staining; 1, weak, light brown staining; 2, moderate, brown staining; and 3, strong, strong brown staining. The staining index (ranging from 0 to 9) was calculated by multiplication of the score for the proportion of LDHA- or LDHB-positive cells by that for the staining intensity. The low- and high-expression groups were defined by staining index ranges of 0–4 and 5–9, respectively (Supplemental Figure 1C).

The αSMA-positive area was measured in the hybrid cell count mode with a BZ-X700 all-in-one fluorescence microscope (Keyence) using a ×4 objective. Samples with αSMA levels greater than the median were considered to have high levels of αSMA, and the αSMA areas were evaluated with respect to patient prognosis.

CD3 and CD8 staining was evaluated by manual counting of the many positive cells within a field of view of the tumor using a ×10 objective.

### Western blot analysis.

Cultured cells were lysed with RIPA buffer containing a protease and phosphatase inhibitor cocktail (Thermo Fisher Scientific). The lysate was sonicated, the debris was removed by centrifugation at 10,000*g* for 10 minutes at 4°C, and the supernatant was collected as a whole-cell lysate. Protein samples were subjected to SDS-PAGE, transferred to PVDF membranes, and blotted with primary antibodies in Can Get Signal Solution 1 (Toyobo) at 4°C overnight. The signals were detected after incubation with rabbit or mouse secondary antibodies in Can Get Signal Solution 2 at room temperature for 1 hour using an ECL Detection System (GE Healthcare, now Cytiva). The antibodies used for Western blotting are listed in Supplemental Table 3. Band signals were quantified with ImageJ software (NIH). Unedited blot images can be found in the supplemental material.

### Cell proliferation assay.

A growth assay was performed with cancer cell lines using an IncuCyte instrument. PANC-1 or PK8 cells were plated in a 96-well plate at 3.0 × 10^3^ cells in 100 μL per well. Panc02 cells were plated in a 96-well plate at 1.0 × 10^3^ cells in 100 μL per well. The plate was inserted into the IncuCyte instrument for real-time imaging, with 4 fields imaged per well every 6 hours over 3–10 days. Data were analyzed using IncuCyte software, which quantified the percentage of red confluence values.

The cell growth of CAFs was measured using an IncuCyte or a Cell Counting Kit-8 (CCK-8; Dojindo Molecular Technologies) assay according to the manufacturer’s protocols. CAFs were seeded in a 96-well plate at 3.0 × 10^3^ cells in 100 μL per well, and the plate was incubated overnight in a humidified incubator at 37°C with 5% CO_2_. The measurement by IncuCyte was performed as described above. For the CCK-8 assay, each well of the plate was also treated with 10 μL of CCK-8 solution at the indicated time points (0, 24, 48, 72, and 96 hours). The absorbance at 450 nm was measured using a microplate reader after incubation of the plate for 90 minutes. Each experiment was performed in triplicate, and the data are presented as the mean ± SEM.

### Extracellular flux analyzer.

The ECAR (mpH/min) and oxygen consumption rate (pmol/min) were measured with a Seahorse XF24 analyzer (Seahorse Bioscience) following the manufacturer’s instructions. Cells were grown to approximately 70%–80% confluence in complete RPMI 1640 medium, trypsinized, and seeded at 2.0 × 10^4^ cells per well (volume = 100 μL) in an XF24 cell culture microplate 24 hours before the assay. The growth medium was changed to XF assay medium, and the plates were incubated at 37°C in a non-CO_2_ incubator for 1 hour before the assay was started. The plates were then transferred to the XF24 analyzer. ECAR values were calculated before and after 1 sequential addition of 2-DG. All measurements were recorded at set time intervals. The medium and 2-DG were obtained from MilliporeSigma. The other compounds and materials were obtained from Seahorse Bioscience.

### Extracellular lactate concentration measurement.

PANC-1 or PK8 cells were plated in a 24-well plate at 5.0 × 10^4^ cells per well (volume = 500 μL). The cells were incubated with 5% CO_2_ for 48 hours at 37°C with 2.0 μM oligomycin or 5% CO_2_ and 1% O_2_. Lactate measurement was performed using a Lactate Assay Kit-WST (Dojindo) according to the manufacturer’s protocols.

### Cell staining and glucose competition assay.

PDAC cell lines were fluorescently stained with a GFP fluorescent protein vector (Takara Bio), and CAFs were fluorescently stained with a tdTomato fluorescent protein vector (Takara Bio) according to the manufacturer’s instructions.

A total of 1.5 × 10^4^ CAFs and 1.5 × 10^4^ PANC-1 or PK8 cells were directly cocultured in a 24-well plate for 24 hours in complete medium. 2NBDG (200 μM; Peptide Institute) or 2-deoxy-2-2(2-oxo-2*H*-chromen-7-yl)amino-d-glucose (200 μM; Peptide Institute) was added to the plate, and the glucose uptake capacity was evaluated 30 minutes later.

Cell counts were performed with a BZ-X700 all-in-one fluorescence microscope using a ×20 objective.

### Measurement of IL-6 and PIGF in the culture supernatant.

Human CAFs were seeded in a 6-well plate at 1.5 × 10^5^ cells per well and incubated with 5% CO_2_ for 24 hours at 37°C, and the medium was changed to glucose starvation medium with or without 10 mM lactate. The CAFs were then incubated with 5% CO_2_ for 72 hours at 37°C, and the supernatant was collected. The supernatant was stored at –30°C, and the concentrations of IL-6 and PIGF were measured at SRL Inc.

### Measurement of lactate and IL-6 concentrations in mouse tumors.

To measure the levels of lactate and IL-6 in tumors from mice, small pieces of tumor samples were lysed with RIPA buffer supplemented with a protease and phosphatase inhibitor cocktail. The lysate was sonicated followed by centrifugation at 10,000*g* for 10 minutes at 4°C, and the supernatant was used for analyses. Lactate concentration was measured using a Lactate Assay Kit-WST as described above. IL-6 was measured using a LEGEND MAX Mouse IL-6 ELISA Kit (BioLegend). The assay procedures were performed according to the manufacturer’s protocol.

### Small interfering RNA transfection.

LDHA or MCT1 expression was downregulated by transfection of cells with predesigned Silencer Select small interfering RNAs (siRNAs) directed against LDHA (s350 and s351, 4390824, Thermo Fisher Scientific) or against MCT1 (s579 and s580, 4390824, Thermo Fisher Scientific). A nontargeting siRNA (4390843, Thermo Fisher Scientific) was used as the negative control. The concentration of siRNA was set at 5 nM to inhibit LDHA expression to less than 30% of that of control cells. Cells were plated in a 6-well plate at 1.5 × 10^5^ cells in 2.5 mL per well. Twenty-four hours after plating, the cells were transfected with 5 nM LDHA-specific or control siRNA using Lipofectamine RNAiMAX Transfection Reagent (Thermo Fisher Scientific) in accordance with the manufacturer’s instructions. After 48 hours of transfection, the supernatant was removed, and the cells were washed with PBS.

### Flow cytometry.

Cell suspensions were incubated with antibodies (listed in Supplemental Table 1) for 30 minutes on ice, washed with PBS containing 2% FBS, and centrifuged twice at 300*g* for 5 minutes at 4°C, and the cell pellets were suspended in PBS. Flow cytometry was performed with a FACSVerse instrument (BD Biosciences). Flow cytometry data were analyzed using FlowJo 3.3 software (Tree Star).

### DNA and RNA extraction.

Genomic DNA and total RNA were extracted from cultured cells using a QIAamp DNA Mini Kit (QIAGEN) and an RNeasy Mini Kit (QIAGEN), respectively, according to the manufacturer’s instructions.

### RNA sequencing.

RNA sequencing was performed with Liaison Laboratory Research Promotion Center (Kumamoto University) support. The concentration and purity of total RNA were measured by a 2100 Bioanalyzer (Agilent). Samples with an RNA integrity number greater than 8.0 were used for subsequent sequencing. The sequence data were obtained by a NextSeq 500 (Illumina) instrument, and the data were converted to Fastq files. Trim Galore (v0.5.0) was used for quality control, and the filtered reads were mapped to the GRCh38 reference genome using STAR (v2.6.0). RSEM (https://github.com/deweylab/RSEM; commit ID 8bc1e21) was used to calculate transcripts per million mapped reads. A heatmap was created using Morpheus (https://software.broadinstitute.org/morpheus/).

Further details of the experimental methods are described in Supplemental Methods.

### Statistics.

All experiments were performed in triplicate, and the data shown are representative of consistently observed results. Data are presented as the mean ± SEM. A 2-tailed Student’s *t* test was used to compare continuous variables between 2 groups. A 1-way ANOVA followed by Tukey’s multiple-comparison test was used to compare multiple groups. Data were analyzed using JMP (version 9, SAS Institute). *P* values less than 0.05 were considered statistically significant. All *P* values and sample sizes are reported in the figures or figure legends.

### Study approval.

Patient sample collection from resected tumors was approved by the Medical Ethics Committee of Kumamoto University (Approval Number 1291). The participants provided written informed consent. All animal procedures and studies were conducted in accordance with the protocol approved by the Institutional Animal Care and Use Committee at Kumamoto University (Approval Number A2021-107).

### Data availability.

All data are provided in this article or the Supporting Data Values file. RNA-sequencing data were deposited in the DNA Data Bank of Japan Sequence Read Archive (accession number DRA016484).

## Author contributions

FK, HB, and TI conceived and designed the study. FK, TS, KY, AN, JY, YT, HW, TY, TA, K Matsumura, NU, and RI acquired data. FK, TS, and NYY analyzed and interpreted data. FK, TS, and TI wrote, reviewed, and/or revised the manuscript. ON, TY, AY, TA, K Matsumura, NU, RI, LB, LF, XH, FW, K Mima, KI, HH, YY, and YM provided administrative, technical, or material support. HB and TI supervised the study.

## Supplementary Material

Supplemental data

Supporting data values

## Version 1 09/21/2023

In-Press Preview

## Version 2 10/23/2023

Electronic publication
