# Supplementary material for: Cancer-associated fibroblasts reuse cancer-derived lactate to maintain a fibrotic and immunosuppressive microenvironment in pancreatic cancer
Source: JCI Insight. 2023 Oct 23;8(20):e163022. doi: 10.1172/jci.insight.163022 (PMC10619496; doi:10.1172/jci.insight.163022)

Supplementary Materials

METHOD DETAILS

Generation of LDHA knockdown cell lines

Panc02 cells were infected with a lentiviral transduction particle targeting *Ldha* from the MISSOIN shRNA Library (Sigma–Aldrich) according to the manufacturer's protocol. Cells were then treated with puromycin after infection to generate stable LDHA knockdown clones. Knockdown of efficacy was validated by western blotting.

LDHA knockdown-rescue experiments

To rescue the expression of LDHA in the stable LDHA knockdown clones in Panc02, we generated a piggybac expression vector plasmid to overexpress the shRNA-resistant LDHA ORF sequence (Vector Builder, vector ID: VB230220-1513drq). The expression vector plasmid was transfected into Panc02 shLDHA clones with the hyPBase piggybac transposon vector (Vector Builder). Cells were then treated with blasticidin (Wako) after transfection, and LDHA expression was confirmed by western blotting.

Lactate assay performed with glucose starvation medium

RPMI 1640 (no glucose) medium with L-glutamine and phenol red (Fuji Film Wako, Osaka, Japan) supplemented with 10% fetal bovine serum (FBS) was used as the glucose starvation medium. Lactic acid (Fuji Film Wako, Osaka, Japan) was added, and the concentration was adjusted to 5 mM or 10 mM before use.

Transwell assay

To evaluate the crosstalk between PDAC cells and CAFs, cells were cocultured using Transwell inserts (Corning, NY USA). To measure lactate-related protein expression in PDAC cells, PANC-1 or PK8 cells were seeded in the lower wells at a density of 1.5×10^4, and CAFs were seeded at the same density in the upper wells. For the measurement of lactate-related protein expression in CAFs, human CAFs were seeded in the lower wells at a density of 1.5×10^4, and PANC-1 or PK8 cells were seeded at the same density in the upper wells. Cells were incubated with 5% CO_2 at 37°C for 72 h, and total protein was harvested from the cells in the lower wells.

Metabolomic analysis

Human CAFs were pretreated with lactate for 24 hours. Any protein was removed with filtration according to the company's protocol, and the intracellular metabolites of 2×10^6

human CAFs were stored at -80°C after extraction. Finally, the metabolites were measured by Human Metabolic Technology (HMT Yamagata Japan). For isotope tracing experiments, 4 x 10^6 human CAFs were cultured with glucose-free RPMI 1640 supplemented with 10% FBS overnight. The next day, the medium was changed to glucose-free RPMI 1640 supplemented with 10% FBS and 10 mM ^{13}C-labeled lactate (Cambridge Isotope Laboratories, Inc., CLM-1579-0) or nonlabeled lactate. After 24 hours, cells were harvested, and metabolomic analysis was performed at the Clinical and Translational Research Center, Keio University Hospital, Tokyo, Japan.

Lactate and IL6 stimulation assay performed with immune cells

Blood donated by healthy volunteers was processed to isolate PBMCs. PBMCs were seeded in a 96-well plate at 1.0×10^5 cells per well. Then, 10 mM lactate and 500 pg/ml recombinant human IL6 (PeproTech, London, UK) were added, and the cells were incubated with 5% CO2 for 48 h at 37°C.

Quantitative Reverse Transcription-Polymerase Chain Reaction

Quantitative reverse transcription–polymerase chain reaction (qRT–PCR) was performed to determine messenger RNA (mRNA) levels in pancreatic cancer cell lines. Total RNA was

isolated from cells using TRIzol (Thermo Fisher Scientific, Waltham, Massachusetts, USA), and the concentration of purified RNA was measured by comparing the A260 and A280 values using a NanoDrop 2000 spectrophotometer (Thermo Fisher Scientific, Waltham, MA, USA). Complementary DNA (cDNA) was generated from total RNA using a ReverTra Ace qPCR RT kit (Toyobo Co. Ltd, Osaka, Japan) according to the manufacturer's instructions and subsequently used as the template for PCR. qRT–PCR was performed as described previously[1]. Transcript levels were measured in a duplicate set of reactions for each gene. Relative hypoxanthine phosphoribosyltransferase 1 levels were used for normalization. The sequences of the primers used in this study are listed in Table S2.

The Cancer Genome Atlas data analysis

RNA-sequencing data for 177 PDAC patients in The Cancer Genome Atlas (TCGA) database were obtained from the Genomic Data Commons Data Portal (https://portal.gdc.cancer.gov/) in November 2021. The PDAC patients were divided into LDHA-high (n = 89) and LDHA-low (n = 88) groups based on *LDHA* gene expression. The RNA-sequencing data of the LDHA-high and LDHA-low PDAC patients were analyzed to estimate CD8+ T-cell infiltration in the tumor microenvironment using the CIBERSORT tool (https://cibersort.stanford.edu/).

SUPPLEMENTARY FIGURES

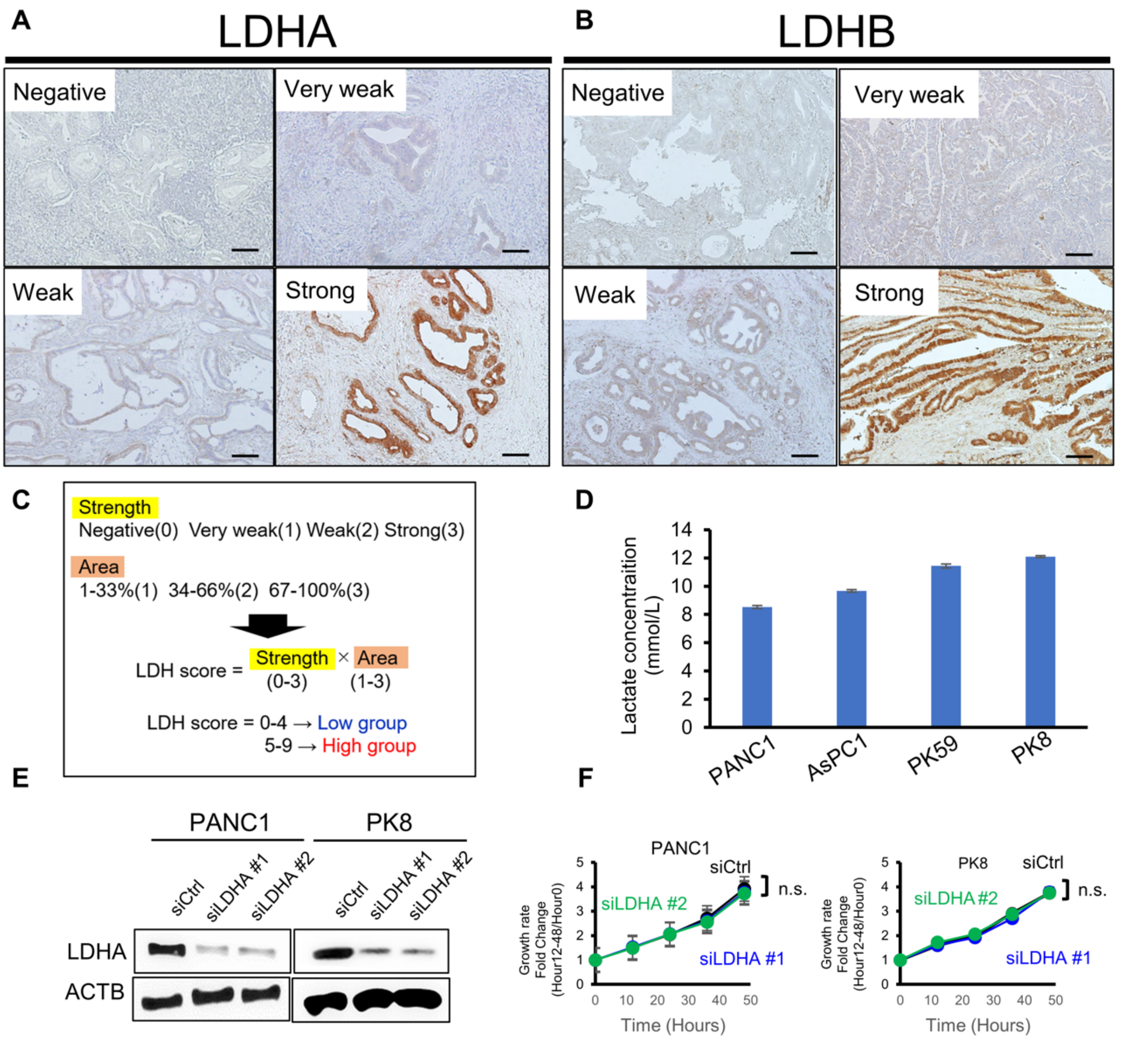

Supplementary Figure 1. IHC evaluation of LDH family proteins in PDAC tumors and *LDHA* gene silencing in PDAC cells. A and B, Representative images of negative (upper left), very weak (upper right), weak (lower left) and strong (lower right) (**A**) LDHA and (**B**) LDHB immunohistochemical staining in resected tumors from PDAC patients. Scale bars, 200 μm. **C**, Evaluation method using the expression strength and area of LDH family member immunohistochemical staining. **D**, Western blot analysis of LDHA expression in PDAC cells

after knockdown of LDHA. **E**, The concentration of lactate in the culture-conditioned medium of PDAC cell lines. **F**, Growth assays performed with PANC-1 and PK8 cells after transfection with siCtrl or siLDHA (n = 3). n.s., not significant.

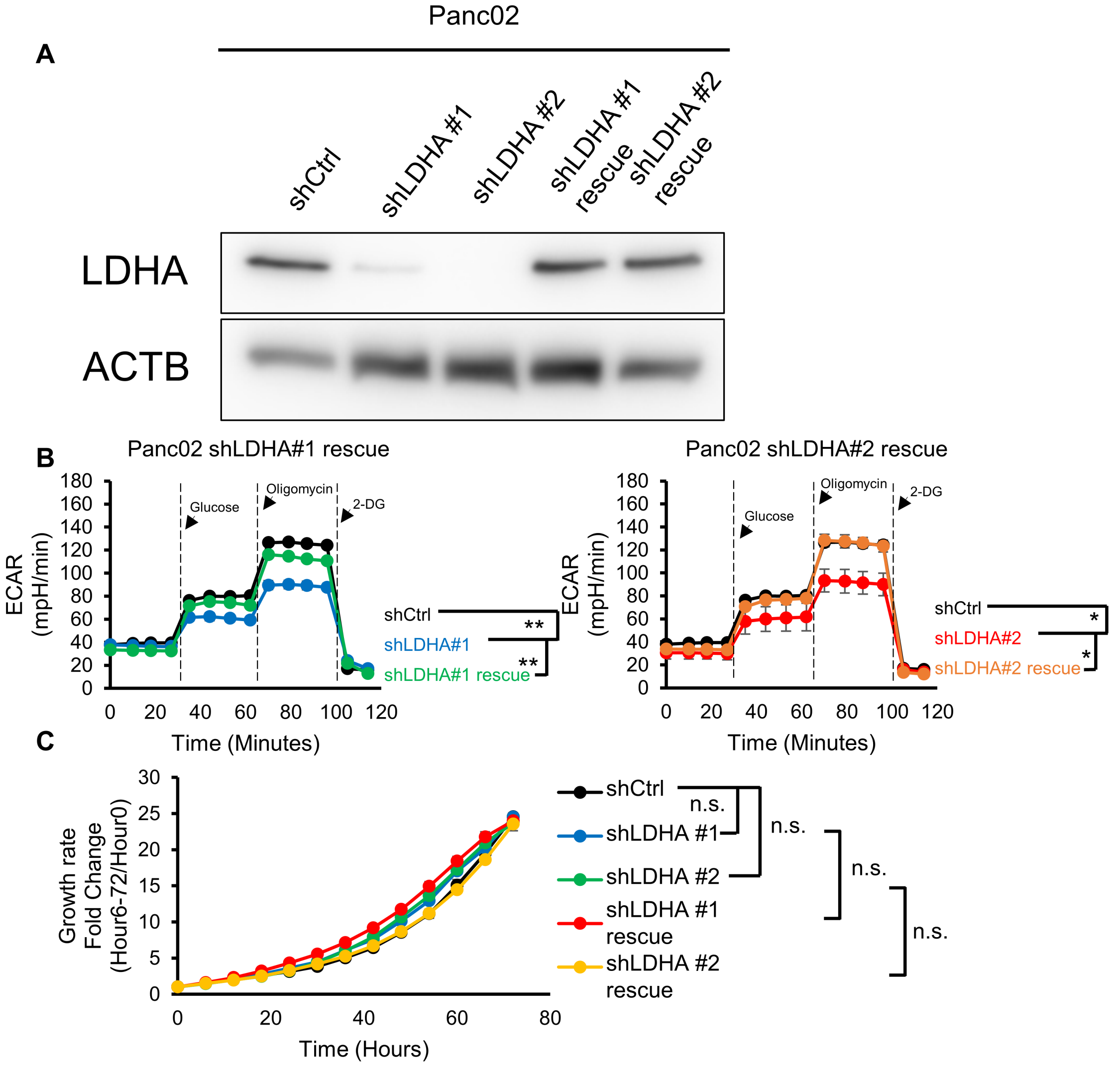

Supplementary Figure 2. Inhibitory effect of LDHA knockout on Panc02 tumor growth.

A, Validation of LDHA knockdown and rescue in Panc02 mouse PDAC cells by western blotting. **B**, Analysis of the glycolytic ability of Panc02 cells using a flux analyzer after LDHA knockdown rescue (n = 3). **C**, Growth of Panc02 cells with LDHA knockdown or LDHA knockdown rescue (n =3). n.s., not significant.

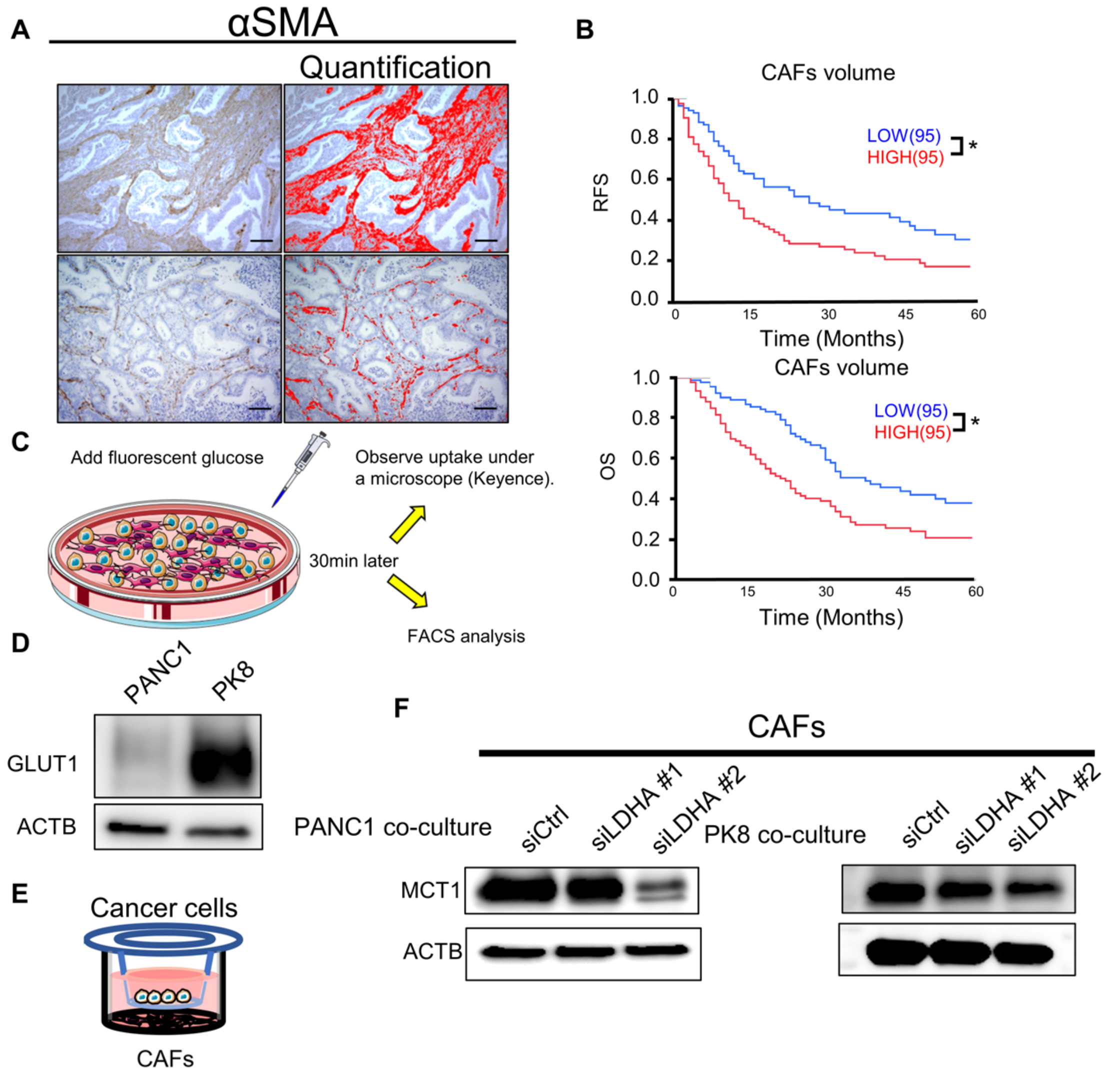

Supplementary Figure 3. Correlation between the amount of CAFs and prognosis in PDAC patients and a glucose competition assay. A, Immunohistochemical staining for αSMA in human PDAC tumors and representative images of αSMA-high (upper left) and αSMA-low (lower left) tumors. The αSMA-positive areas detected by BZ-X700 software are visualized in red in the right panel. Scale bars, 200 μm. **B**, Kaplan–Meier survival analysis of the RFS (upper) and OS (lower) of PDAC patients stratified based on the amount of CAFs, as determined by measuring the αSMA-positive area as in (**A**). **C**, Schema for the glucose

competition assay. **D**, Western blot analysis of GLUT1 expression in PANC1 and PK8 cells. **E**, Schematic showing the coculture of cancer cells and human CAFs using transwells. **F**, Western blotting analysis of MCT1 expression in CAFs cocultured with LDHA knockdown PANC1 or PK8 cells. *, $p < 0.05$.

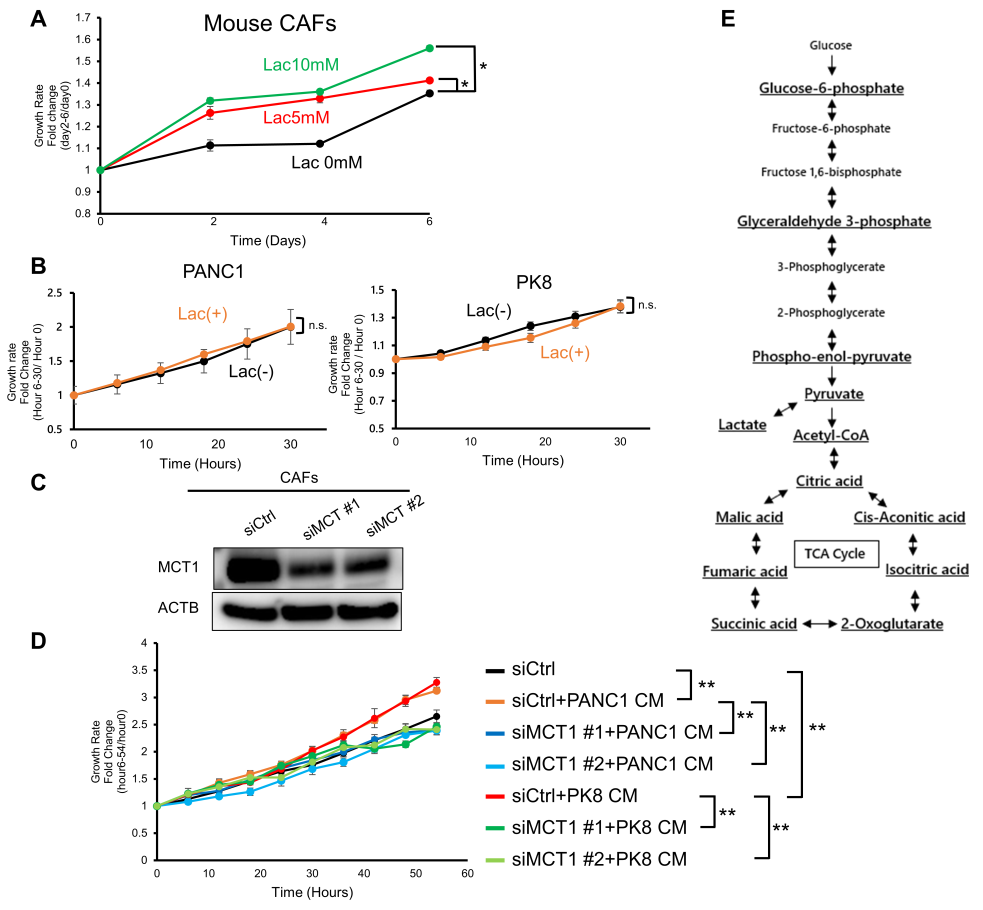

Supplementary Figure 4. CAFs use lactate as a fuel via the TCA cycle. A, Growth assay performed with mouse CAFs with or without lactate in glucose-free medium (n = 3). **B**, Growth assay performed with PANC-1 (upper) or PK8 (lower) cells with or without lactate in glucose-free medium (n = 3). **C**, Validation of MCT1 knockdown in CAFs by western blotting. **D**, Growth assay performed with MCT1 knockdown human CAFs treated with culture conditioned medium from PANC1 or PK8 cells. **E**, Schematic showing the route map by lactate metabolism. *, $p < 0.05$; **, $p < 0.01$; n.s., not significant.

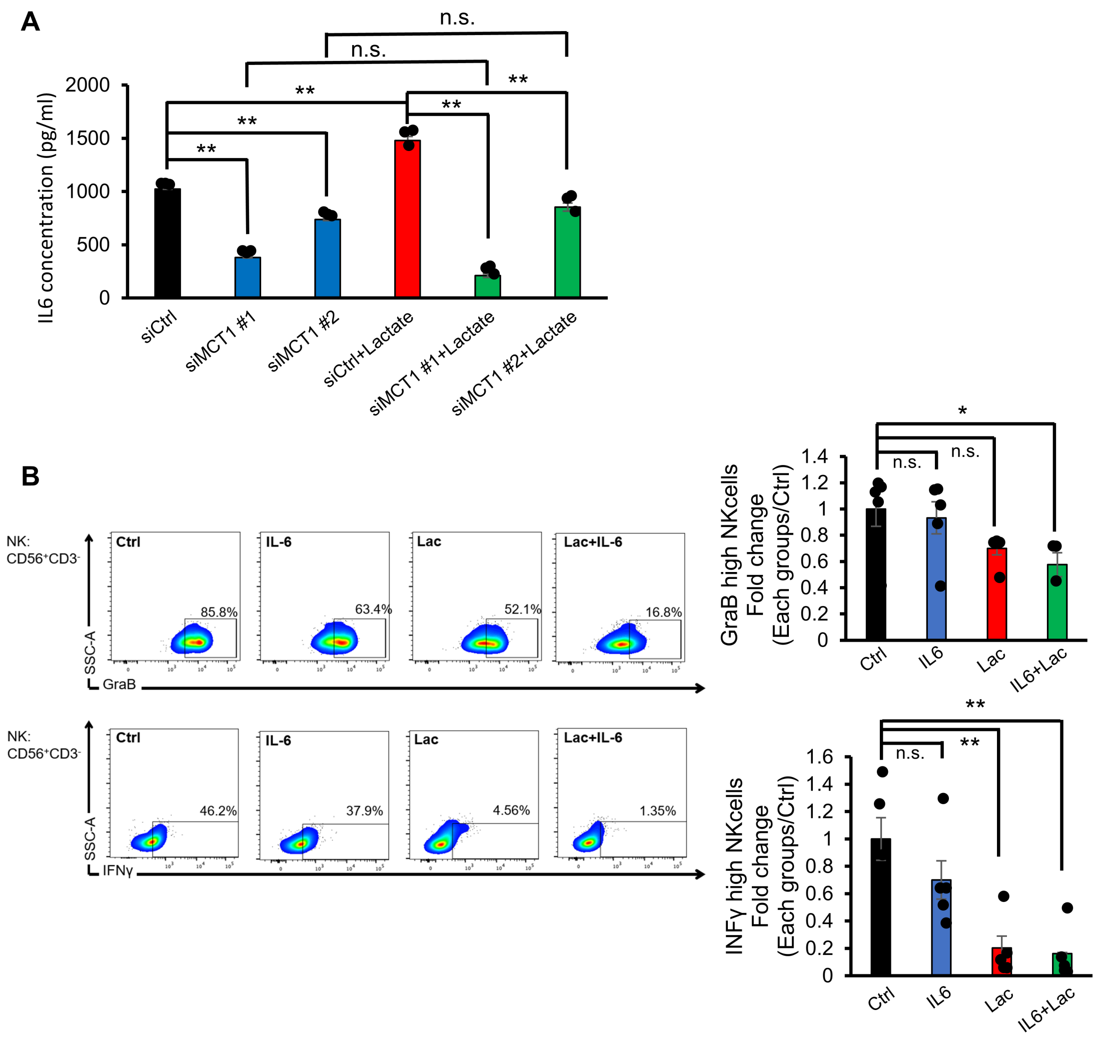

Supplementary Figure 5. Lactate-stimulated CAFs produce IL6 and suppress antitumor immunity. A, Quantification of the IL6 concentration in the conditioned medium of MCT1 knockout CAFs stimulated with or without lactate (n = 3). **B**, Flow cytometric analysis of IFNγ expression in NK cells in PBMCs stimulated with IL6 or lactate alone or IL6 and lactate (n = 3). *, p < 0.05; **, p < 0.01; n.s., not significant.

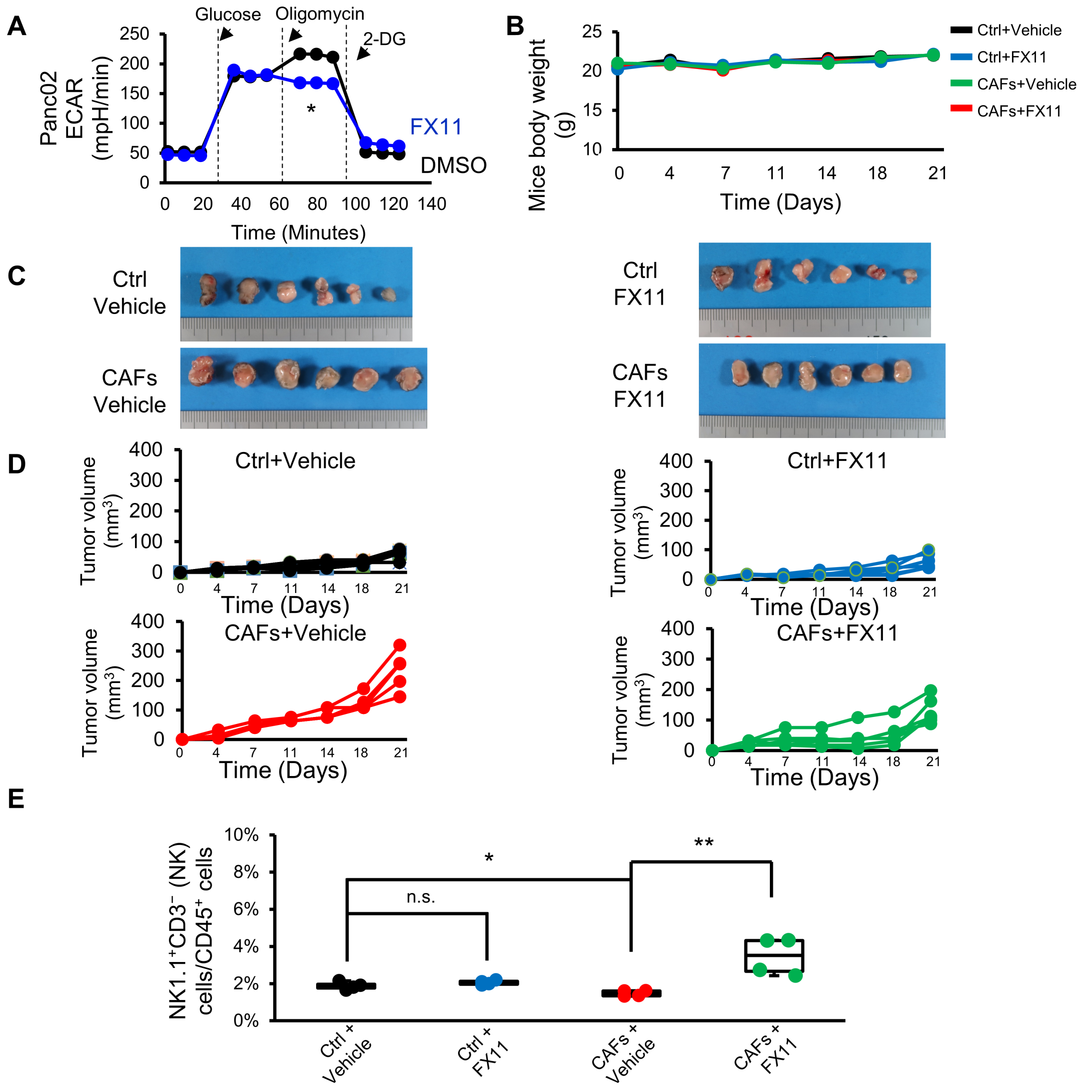

Supplementary Figure 6. FX11 suppresses the growth of CAF-rich PDAC tumors. A, Analysis of the glycolytic ability of Panc02 cells treated with FX11 using a flux analyzer (n = 3). **B**, Body weight curves of mice in the Ctrl or CAF group treated with vehicle control or FX11 (n = 6). **C**, Gross images of the tumors from the Ctrl and CAF groups treated with vehicle control or FX11 resected on day 21 (n = 6). **D**, Tumor growth curves of individual mice in the Ctrl group treated with vehicle control (upper left), Ctrl group treated with FX11 (upper right), CAF group treated with vehicle control (lower left), and CAF group treated with FX11

(lower right) (n = 6). **E**, Quantification of the percentage of INFγ$^+$ cells in CD3$^+$NK1.1$^+$ NK cells in tumors from each group analyzed by flow cytometry (n = 4). *, $p < 0.05$; **, $p < 0.01$; n.s., not significant.

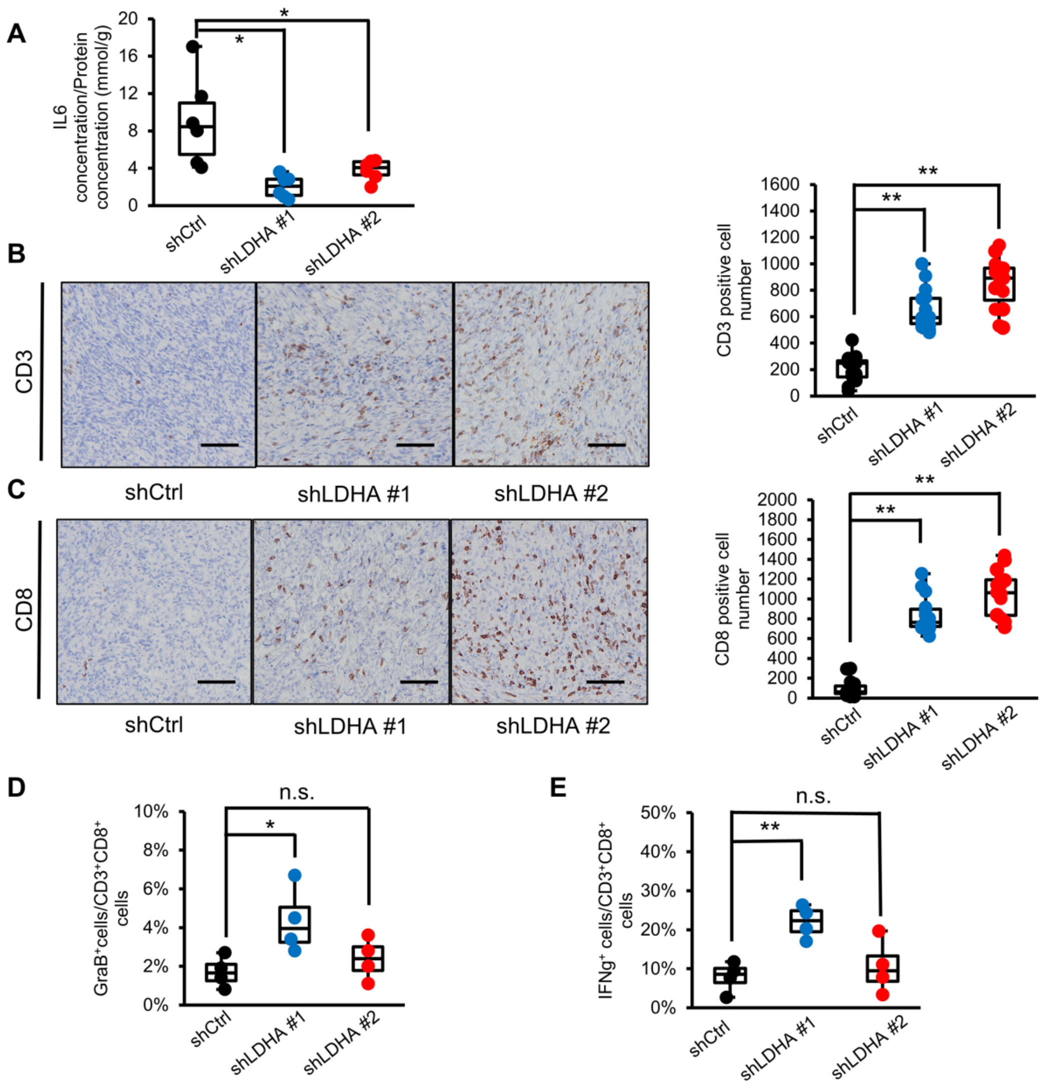

Figure 7. LDHA inhibition ameliorates the fibrotic and immunosuppressive TME in orthotopic PDAC mouse models. A, Measurement of the IL6 concentration in the lysates from shCtrl or shLDHA tumors in the orthotopic mice models (n =6). **B** and **C**, Representative images of (**B**) CD3 and (**C**) CD8 immunohistochemical staining and quantification of the number of (**B**) CD3- or (**C**) CD8-positive lymphocytes in tumors from shCtrl or shLDHA tumors (n =6). **D** and **E**, Flow cytometric analysis for quantification of the percentages of (**B**) $GraB^+$ cells in $CD3^+CD8^+$ T cells and (**C**) $IFN\gamma^+$ cells in $CD3^+CD8^+$ T cells infiltrated into shCtrl or

shLDHA Panc02 tumors (n = 4). *, $p < 0.05$; **, $p < 0.01$; n.s., not significant.

Table S1. Summary of information on the cells used in this study.

	PANC1	AsPC1	PK59	PK8
Species of origin	Human	Human	Human	Human
Sex of cell	Male	Female	Female	Unspecified
Age at sampling	56Y	62Y	66Y	Unspecified
Disease	Pancreatic ductal adenocarcinoma	Ascites from Pancreatic ductal adenocarcinoma	Liver metastasis from Pancreatic carcinoma	Liver metastasis from Pancreatic ductal adenocarcinoma
Genetic mutation	CDKN2A deletion<br>KRAS mutation<br>TP53 mutation	MAP2K4 deletion<br>CDKN2A mutation<br>KRAS mutation<br>SMAD4 mutation<br>TP53 mutation	KRAS mutation	KRAS mutation<br>TP53 mutation
Histotype	Epithelial-like	Epithelial-like	Epithelial-like	Epithelial-like

Table S2. Sequences of the primers and probes used for qPCR analysis.

Animal species	Official Symbol	Alias	Forward Primer	Reverse Primer
Human	ACTB	β-actin	attggcaatgagcggtt	cgtggatgccacaggact
Human	IL6		caggagcccagctatgaact	gaaggcagcaggcaacac
Human	PIGF		ctcgggacgtctgagaagat	cagcagacaaggcccact
Mouse	Actb	β-actin	ccaaccgtgaaaagatgacc	accagaggcatacagggaca
Mouse	Ldha		ggcactgacgcagacaag	tgatcacctcgtaggcactg
Mouse	Il6		acaagtccggagaggagact	ttgccattgcacaactcttttc

Table S3. Antibodies used in this study.

	Target	Manufacturer	Catalog number	Dilution
Primary	LDHA/LDHC	Cell Signaling Technology	#3558	1:1000 (WB)<br>1:100 (IHC)
	MCT1	Abcam	ab85021	1:1000
	ACTB	Cell Signaling Technology	# 4967	1:1000
	CD3	Abcam	ab16669	1:100
	CD8	Abcam	ab209775	1:200
	αSMA	Abcam	ab5694	1:1000
	GLUT1	Abcam	ab40084	1:1000
Secondary	Anti-rabbit IgG-HRP	Cell Signaling Technology	# 7074	1:5000
	Anti-mouse IgG-HRP	Cell Signaling Technology	# 7076	1:3000

References

1 Ishimoto T, Miyake K, Nandi T, Yashiro M, Onishi N, Huang KK *et al*. Activation of Transforming Growth Factor Beta 1 Signaling in Gastric Cancer-associated Fibroblasts Increases Their Motility, via Expression of Rhomboid 5 Homolog 2, and Ability to Induce Invasiveness of Gastric Cancer Cells. *Gastroenterology* 2017; 153: 191-204.e116.

Full unedited blot for Figure 3G

MCT1

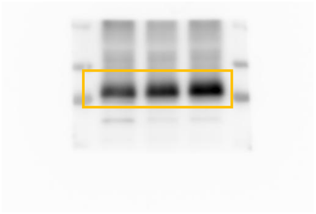

ACTB

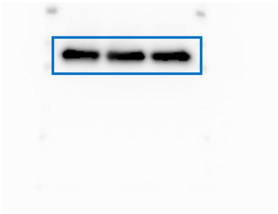

Full unedited blot for Supplemental Figure 1D

PANC1 LDHA

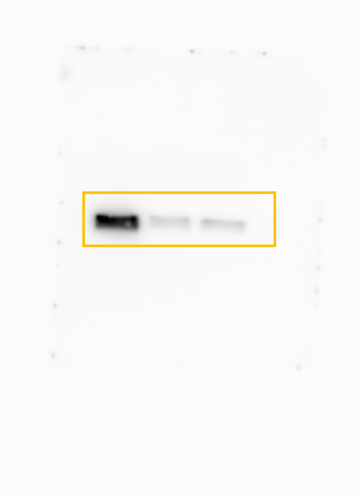

PK8 LDHA

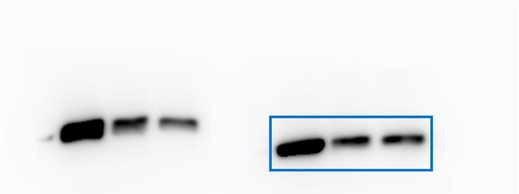

PANC1 ACTB

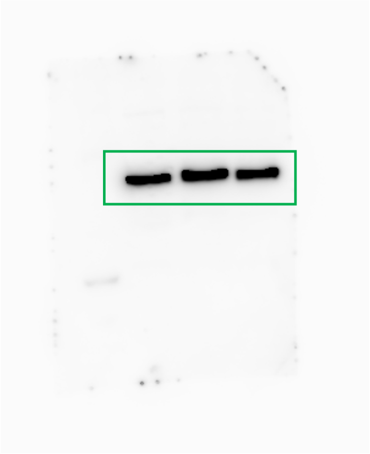

PK8 ACTB

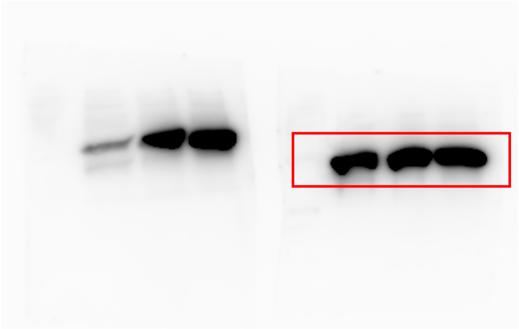

Full unedited blot for Supplemental Figure 2A

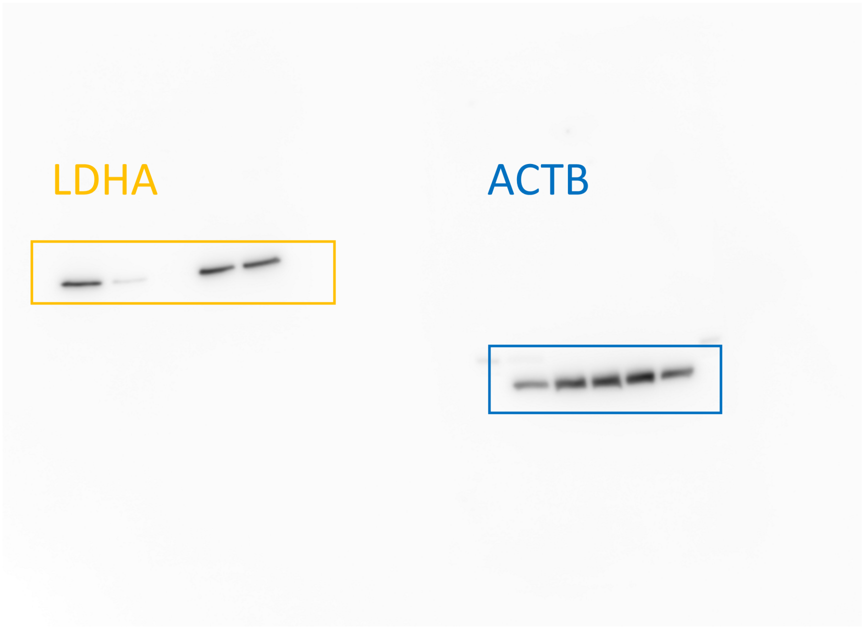

Full unedited blot for Supplemental Figure 3D

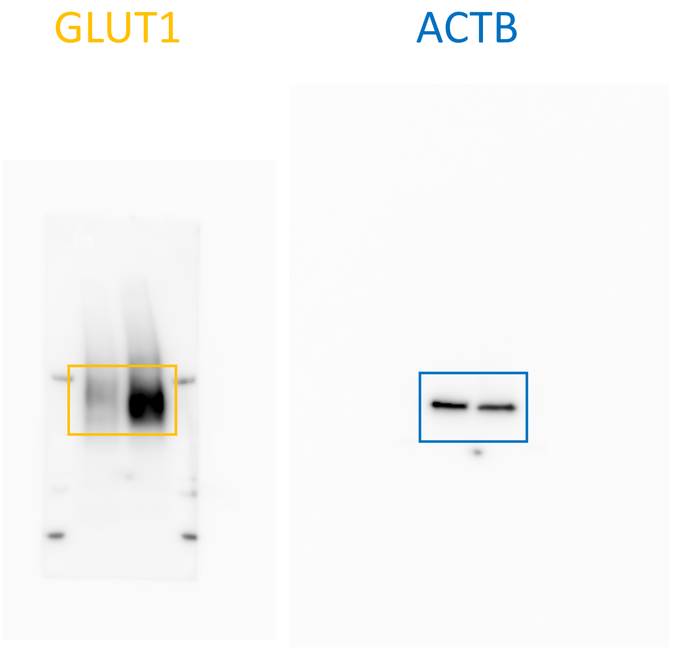

Full unedited blot for Supplemental Figure 3F

CAFs PANC1 co-culture MCT1

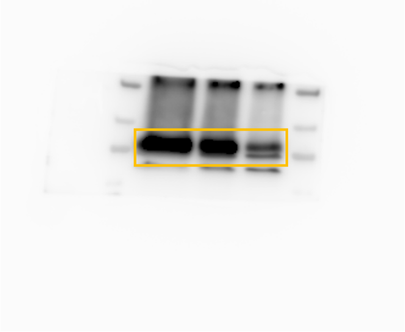

CAFs PK8 co-culture MCT1c

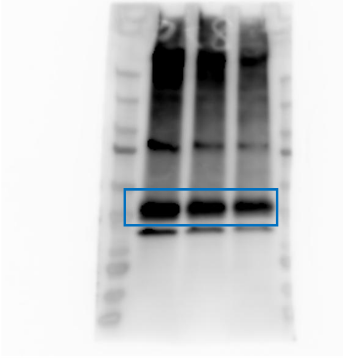

CAFs PANC1 co-culture ACTB

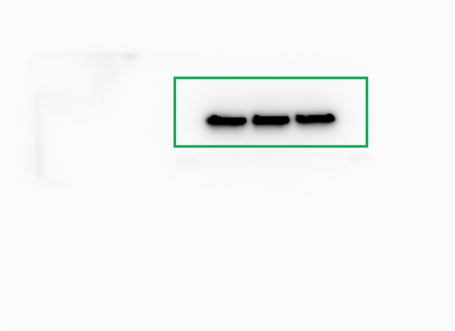

CAFs PK8 co-culture ACTB

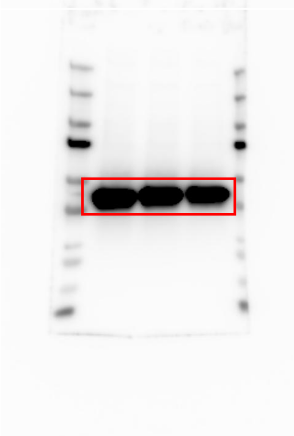

Full unedited blot for Supplemental Figure 4C

MCT1

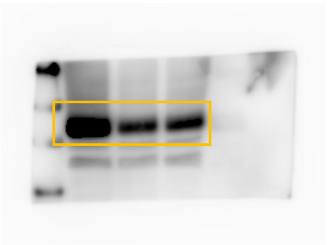

ACTB

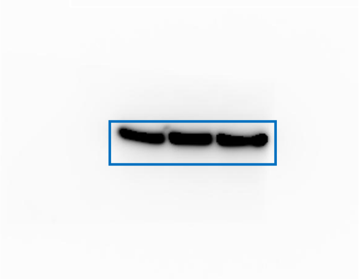

Supplement: Supplemental data [file jciinsight-8-163022-s105.pdf]
